# A dual-process approach to cooperative decision-making under uncertainty

**DOI:** 10.1371/journal.pone.0265759

**Published:** 2022-03-22

**Authors:** Daniela Costa, Joana Arantes, José Keating

**Affiliations:** Psychology Research Center, University of Minho, Braga, Portugal; Universidad Carlos III de Madrid Departamento de Matematicas, SPAIN

## Abstract

Cooperative behaviors are typically investigated using social dilemmas inserted into scenarios with well-known characteristics. Nonetheless, in real life, group members may be uncertain about what others will decide (social uncertainty) and the characteristics of the dilemma itself (environmental uncertainty). Previous studies have shown that uncertainty reduces the willingness to cooperate. Dual-process approaches to cooperation have given rise to two different views. Some authors argue that deliberation is needed to overrule selfish motives, whereas others argue that intuition favors cooperation. In this work, our goal was to investigate the role of intuitive mental processing on cooperation in a prisoner’s dilemma game involving uncertainty. Our results showed that participants cooperated less with their counterparts as the number of rounds progressed, suggesting a learning process and that intuitive mental processing in the first 50 rounds appears to favor cooperation under both deterministic and stochastic conditions. These results may help clarify the literature’s mixed effects regarding cognitive processing manipulation on cooperation. Developing a better understanding of these effects may improve strategies in social problems involving cooperation under uncertainty and cognitive constraints.

## Introduction

Cooperative behaviors are defined as actions in which individuals benefit another individual or group of individuals while having a personal cost to themselves [1]. These types of behaviors have existed since the days of our hunter-gatherer ancestors, being demonstrated through various actions such as joint living, cooperative hunting, and sharing resources, all of which are necessary for their survival [2]. Cooperation continued to change and evolve within small and large societies alike throughout the centuries and gave the human species an enormous evolutionary advantage [2]. Still, generalized cooperation remains one of the greatest unresolved mysteries in the evolution of our species [3,4]. While many individuals recycle, give blood, pay taxes, vote, tip, and donate to charities in the modern world, not all individuals choose to cooperate [5]. Thus, the question emerges: why do some individuals cooperate while others do not?

Several mechanisms have been proposed to explain cooperation [6,7], such as (i) kin selection (helping relatives [8]); (ii) direct reciprocity (trading favors [9]); (iii) indirect reciprocity (maintaining a good reputation [10,11]); (iv) special selection (clusters of cooperators outcompete defectors [12,13]); (v) multilevel selection (competition is not only between individuals but also between groups [14]); and (vi) enforced cooperation (mechanism for rewarding cooperators or punishing defectors [4,15]).

Cooperative behavior has mainly been investigated using social dilemmas [16–18]. In the beginning, social dilemmas were characterized by two essential properties [19]:

(i) each individual receives a higher payoff if they decide to defect, no matter what the others choose, and (ii) all individuals receive a lower outcome if all defect rather than if all cooperate. Later, social dilemmas were defined as interdependence situations characterized by a conflict between immediate self-interest and long-term collective interest [18].

Researchers have studied social dilemmas from different disciplines and used different methodologies to understand cooperation [20]. Two of the most prominent and highly used social dilemmas are the Prisoner’s Dilemma (PD) and the Public Goods Game (PGG) [21–23]. For example, in the PGG dilemma, all members may benefit regardless of whether they contributed to the good [20]. The PD has received much attention throughout the years and has become a leading paradigm for exploring cooperative decision-making [21,24–26]. It is often used as the basis for defining a resource dilemma [18]. Specifically, this game involves two players, and each player needs to choose between two strategies: cooperation (C) or defection (D—see Table 1). The relative value of the four possible outcomes defines this game [17,21]. Both players will receive a reward (*R)* if both cooperate and a punishment (*P*) if both choose to defect. However, when a defector meets a cooperator, they exploit the cooperator and receive the temptation (*T)*, and the cooperator is left with the *sucker’s payoff* (*S)*. In the prisoner’s dilemma game, the outcomes should have the following relationship: *T*>*R*>*P*>*S* [26,27] Even though it is individually optimal to defect (no matter what the other does), the optimal result, assuming none of the parties is willing to be exploited, requires both parties to cooperate [21,26].

**Table 1:** Payoff matrix of a Prisoner’s Dilemma (PD) game.

		Player 2	
		C	D
Player 1	C	R, R	S, T
	D	T, S	P, P

These social dilemmas have been used to model human behavior in social dilemmas like pollution control, intergroup conflict, or the depletion of natural resources [20].

### Cooperation under uncertainty

In most social dilemma experiments, the dilemma’s characteristics (e.g., the payoff matrix, the number of iterations, and the number of participants) are usually known by all members with certainty [28]. However, such defining characteristics are not always evident in real-life situations. For example, several alternative strategies may be available in real-life situations, and the outcome of interactions may be uncertain [29]. Such uncertainty may be fractioned into two distinct constructs: risk (i.e., the probability of outcomes are known) and ambiguity (i.e., the probability of outcomes are unknown [30]). In uncertain social dilemmas, members of a group may be unsure about what the other players will decide (i.e., social uncertainty), as well as about the characteristics of the dilemma itself (i.e., environmental uncertainty [28,31]). Several studies have shown that environmental and social uncertainty reduces the individuals’ willingness to cooperate in various social dilemmas [18,28,32,33]. In fact, individuals tend to avoid uncertainty both within social [34] and non-social domains [35,36].

Nonetheless, some studies have shown that if the uncertainty is lowered, providing participants some feedback regarding how their counterparts responded in the previous trial, their willingness to cooperate might increase [37,38]. If individuals find that their counterparts cooperate with them, they will also be more willing to cooperate. Conversely, if their counterparts did not cooperate, they will become less motivated to cooperate in subsequent trials [28,39]. A recent framework explains that humans, to reduce social uncertainty, tend to use three interrelated mechanisms [34]: i) *automatic inferential processes* like the formation of impressions that rapidly and with little effort narrow one’s predictions using past knowledge and contextual cues for reducing the uncertainty of how the other person might think and behave; ii) more *controlled inferential processes* that additionally adjust these automatic predictions through a mental representation of the other person’s thoughts and feelings; and iii) *learning processes* that update one’s predictions based on feedback.

Several studies have investigated how different types of environmental uncertainty (e.g., resource size uncertainty, group size uncertainty) influence cooperation [28]. For instance, resource size uncertainty (i.e., the payoff matrix) decreases the overall cooperation rate [28]. Indeed, previous studies have observed that participants tend to: (i) overestimate the resource pool’s size; (ii) increase their requests and overharvest and also expect others to increase their request [33,40–43]; and (iii) there is an increase of the inter-participant variance [28,43].

Additionally, Gong et al. examined the degree of cooperation both in individuals and groups under resource size uncertainty using two versions of the PD: one Stochastic Prisoner’s Dilemma (SPD) and one Deterministic Prisoner’s Dilemma (DPD) [44,45]. On the SPD, the outcomes of strategies are uncertain with known probabilistic outcomes. Each player knows that cooperation without their opponent’s corresponding cooperation exposes them to losses with defined probabilities. The related deterministic game is obtained by replacing the stochastic game’s probabilistic outcomes with the expected values (value x corresponding probability). Although groups are less cooperative than individuals in the DPD game, a reversed effect was observed when uncertainty was present. Groups were more cooperative in SPD games than individuals [44]. The authors report a learning process in their study as participants repeatedly played a game with feedback. The data showed that participants tended not to cooperate as the number of rounds and supergames increased. Other studies also report that cooperation significantly decreased over time under repeated games with feedback [2,46].

Considering now another type of environmental uncertainty, group size, previous studies have shown a more positive effect on cooperation [47–52]. This type of uncertainty leads to more contributions in resource dilemmas [49] and decreases over-harvesting compared to group size certainty[47]. In a recent study, the authors manipulated the group size under linear public good games and found that uncertainty/risk significantly positively affected conditional cooperation [51].

Even though many researchers have explored the influence of uncertainty on social decision-making, this topic remains poorly understood [30]. Moreover, research still needs to explain why and how cooperation decreases in some types of social and environmental uncertainty. Thus, to better understand the influence of risk and uncertainty on cooperation, more studies should be developed to identify how the uncertainty constructs and cognitive mechanisms influence cooperation [30].

### A dual-process approach to cooperation

The cognitive mechanisms underlying cooperative behavior are explored in the literature by applying dual-process models, which conceptualize decisions as arising from a competition between intuitive ("automatic") versus deliberative ("controlled") cognitive processes [53]. Knowledge of dual-process theories has increased over recent years [53–58]. One of the models developed assumed two types of processes: Type 1 and Type 2 [57]. Type 1 processing is characterized by having autonomy–it does not require "controlled attention." Autonomous processes tend to be fast, automatic, nonconscious, independent of cognitive ability, experience-based decision-making, and do not put a heavy load on central processing capacity [57]. In this definition, Type 1 processes give rise to intuitions [59,60]. In Type 2 processing, responses rely on hypothetical thinking and working memory. This type of response is slow, conscious, sequential, consequential decision making and correlated with cognitive ability [57].

Researchers have manipulated cognitive processes using time constraints methods [54,61,62]. For example, to promote Type 1 processing, participants respond within a given time window ("time pressure" condition). In contrast, to facilitate Type 2 processing, participants may think carefully for some time over the decision problem before deciding ("time delay" condition) [54,61,62].

The seminal study regarding decision time and cooperation revealed that time pressure manipulation increases cooperative behavior relative to the time delay in the one-shot public goods game [61]. Subsequent studies went on to replicate these findings and found that this effect increases significantly amongst inexperienced and trusting participants compared with individuals experienced in experimental economic games [63,64]. This observation led to the development of a theory that predicts when intuition will, and will not, influence cooperation–The Social Heuristics Hypothesis (SHH) [64,65]. According to the SHH, "people internalize strategies that are typically advantageous and successful in their daily social interactions" [64]. Researchers have distinguished between pure and strategic cooperation to understand better SHH effects [54,62]. Pure cooperation refers to situations where defection is always payoff maximizing, regardless of the other person’s decision (e.g., one-shot prisoner’s dilemma).

On the other hand, strategic cooperation refers to how cooperation or defection can maximize payoff, depending on the other players’ decisions [54,62]. In repeated interactions (e.g., repeated prisoner’s dilemma), cooperation can be payoff-maximizing as long as the other players’ reputation is known and the possibility of reciprocation exists [66,67]. If deliberation favors payoff-maximizing responses, then promoting deliberation should only reduce cooperation when defection is the payoff-maximizing choice. Therefore, intuition should favor cooperation when defection is payoff-maximizing, while deliberation should favor defection [62,68]. The SHH assumes that over time, strategies that are typically successful become the default response. Thus, individuals who generally interact in environments where cooperation is advantageous should be predisposed to cooperate even if, on occasion, this does not maximize outcomes: they have internalized a cooperative strategy [62,63,66,69]. On the other hand, deliberation adjusts behavior towards the optimum strategy for a given situation. Thus, deliberative responses tend to be less cooperative than intuitive responses in interactions where defection is the optimum strategy [62].

Several subsequent studies using PGG have obtained results consistent with the seminal work, revealing a positive effect of time pressure on cooperation [70]. Moreover, this result was found even when the game is played with outgroup members [71,72] and in competitive public goods games [73].

Other studies using PD and PGG found the opposite effect of time pressure on cooperation [74–76]. Furthermore, several studies have also failed to find the effect of time pressure on cooperation [69,77–79]. Moreover, several recent studies have found that individual factors have a moderator effect on time pressure on cooperation. For example, a recent study found no overall effect of time pressure on cooperation in both one-shot and repeated PGG; however, they found that time constraints interact with two individual characteristics: Social Value Orientation and Strategic Uncertainty [5]. Additionally, while exploring the moderating effect of individual risk factors (integrating measures of individual dominance, self-control, and self-construal), the authors found that time pressure increases cooperation, but only among low-risk propensity men [80]. However, the effect reverses among high-risk propensity men [80].

Contrarily to previous findings that cooperation is faster than defection, or vice versa, a study has shown that reciprocal decisions occur more quickly in repeated interactions [81]. Furthermore, the authors show that cooperation is faster than defection in cooperative social environments, while defection is faster than cooperation in non-cooperative environments. The result found can be explained because, in repeated interactions, people are strongly influenced by the previous behavior of their interaction partners [82,83]. However, in the study, the authors also evaluate the situation when participants do not know the decision of their interaction partners (e.g., in an unknown environment). In this situation, the decision times are similar to those in the cooperative environment–cooperation is faster than defection. Results from the unknown environment are consistent with the idea of the SHH that participants’ frequent interactions with cooperative institutions caused them to expect others to cooperate and to want to cooperate themselves [81].

The first meta-analyses found evidence supporting the SHH’s prediction; intuition increases cooperation in games where non-cooperation was strictly payoff-maximizing [62]. On the other hand, results found that intuition does not affect games where cooperation could be payoff-maximizing (e.g., games in which reciprocity was possible) [62]. Moreover, a recent meta-analysis found that the effect of intuition on cooperation was driven by conceptual primes explicitly asking people to rely on their emotions [84]. Besides, in a response paper to a pre-print version of the meta-analysis, the author found a positive effect of intuition on a one-shot game, which remains significant when restricting the analysis to studies that do not use explicit primes [68].

### The present study

The present study aims to empirically investigate the cognitive mechanisms of human cooperation by analyzing the impact of intuition on individual cooperation under uncertainty. Given that this is a new research field and that the uncertainty present in our lives can affect our judgments and decisions, studying these two variables–cognitive mechanisms of cooperation and uncertainty- becomes essential.

Additionally, observing the choices of subjects over time is crucial once real-life situations are often characterized by repeated interactions [1]. Furthermore, extending research on decision time to repeated games may explain the mixed results found in the literature and may help to clarify the relationship between decision time and cooperation [54,81,85]. Specifically, in this study, we will examine how rates of cooperation change 1) during the repeated prisoner’s dilemma and 2) between different combinations of time pressure and uncertainty.

Following previous observation regarding the decrease in cooperation in the repeated prisoner’s dilemma [44], we expect cooperation to decrease over time in our first hypothesis. Given this pattern of cooperation rates and the findings in games with repeated interactions [81] in our second hypothesis, we expect that time pressure manipulation will favor cooperation in the first interactions since we expect a more cooperative environment in the beginning. On the contrary, in our third hypothesis, we expect that time pressure manipulation will not affect cooperation as the game progresses.

Additionally, we aim to understand the effect of the manipulation of uncertainty on individual cooperation, considering that previous findings on uncertainty stated that uncertainty reduces the willingness to cooperate [28,44]. Therefore, our fourth hypothesis is that cooperation will be higher in the deterministic version of the PD, and individuals will be less cooperative in the stochastic version of the game (when uncertainty is present).

To extend the knowledge in this field, we also aim to understand the relationship between uncertainty and time pressure manipulation since cooperative behavior diminishes under environmental uncertainty situations [44] and under time delay [62,64]. However, when adding a time delay, cooperative behavior will decrease under conditions of uncertainty. Therefore, we hypothesized that time pressure manipulation augments cooperative behavior under conditions of uncertainty.

## Method

### Participants

A power analysis using the G*Power computer program (version 3.1) [86] indicated that a total sample of 102 participants would be needed to detect significant effects of the uncertainty effect with 90% power using a z test with a binomial distribution and alpha .05. Therefore, our sample comprised a total of 112 university students (88 women), ranging between 17 and 44 years of age (*M* = 21.90, *SD* = 4.83). Participants belonged to the University of Minho, with 107 psychology students. Regarding the year of the degree they are attending, sixty-nine participants (61.6%) reported being in a bachelor’s degree, forty-seven participants (25.0%) report being in the masters, and fifteen participants (13.4%) report being in the first year of a doctoral degree or doing a postgraduation. From the total number of participants, one hundred four participants (92.9%) were Portuguese, five Brazilian, two Spanish, and one British. However, all students report living and studying in Portugal. Thirty-three participants (29.5%) reported a medium-high socio-economic value, sixty-four participants (57.1%) reported a medium value, and fifteen (13.4%) reported a medium-high socio-economic value. All participants gave their written informed consent, according to the Helsinki Declaration. The Ethics Committee for Research in Social and Human Sciences (CEICSH) of the University of Minho, approved the study. There were no exclusion criteria. Participants were naive concerning the whole experimental procedure (they believed they were playing a problem-solving experiment) and received credits for their involvement. Two participants were randomly chosen to receive a prize using the same procedure as Gong et al. [44]; however, instead of money, participants received a shopping voucher proportional to the points gained in the experiment.

### Materials

#### Sociodemographic questionnaire

Participants answered questions regarding age, gender, nationality, socio-economic level, and university degree level.

#### Balloon Analogue Risk Task (BART;[87])

This task measures risk-taking propensity and comprised 30 balloon trials. In this task, the computer screen showed: i) a computerized balloon; ii) the number of pumps on that balloon; iii) a second display listing the points earned on that balloon, labeled "*Points so far in this balloon*"; and iv) a permanent points-earned display, labeled "*Total Points*", that added up the points scored on the 30 balloons presented. Each time participants pressed the e key on the keyboard (labeled "*e*" for a pump), the balloon could expand (about 0.125 inches [0.3 cm] in all directions), and consequently, participants would earn 10 points. After pressing the "*e"* key as many times as participants wanted, they needed to press the "*p*" key on the keyboard to stop and collect the points. However, the balloon could explode when participants were pressing the "*e*" key for a pump, and consequently, participants would lose all the points earned on that trial. After each trial, a fixation cross appeared on the center of the screen for 1s, and the subsequent balloon trial began. The probability that a balloon would explode ensured that, on average, the optimal number of pumps in each trial was 64. Briefly, 15 integers between 1 and 128 were randomly generated. These random numbers determined the explosion points for 15 balloon trials. The remaining 15 trials are set equal to 128—X + 1, where X was the vector of 15 randomly generated. Before starting the task, participants played one practice trial to ensure they understood the terms.

#### Big Five Inventory (BFI; [88,89])

This instrument assesses personality traits in five dimensions: agreeableness, conscientiousness, extroversion, neuroticism, and openness to experience. It comprises 44 easy-to-understand short sentences, each referring to only one of the Big Five personality dimensions. Participants indicate the extent to which each trait applies to themselves (e.g., "*I see myself as someone who tends to be lazy*"), using a 5-point scale ranging from 1 "*Strongly disagree*" to 5 "*Strongly agree*". The BFI has good internal reliability (Cronbach’s α = 0.65–0.86) [89].

#### Interpersonal Reactivity Index (IRI;[90,91])

This instrument assesses empathy and comprises 24 statements about feelings and thoughts that the person may or may not have experienced. The instrument contains four subscales, each with six statements: Perspective Taking (PT), Empathic Concern (EC), Personal Discomfort (PD), and Fantasy (F). Participants indicate, for each statement, the extent to which it applies to themselves (e.g., " *I often have tender*, *concerned feelings for people less fortunate than me*") on a 5-point Likert scale, from 0, *"It does not describe me well"*, to 4, "*Describes me very well*". The translation and validation study to the Portuguese population confirmed the adequate internal consistency and good reliability of this instrument in the assessment of empathy (PT Cronbach’s α = 0.73; EC Cronbach’s α = 0.76; PD Cronbach’s α = 0.80; F Cronbach’s α = 0.84) [91].

#### Short-form of the Positive and Negative Affect Schedule (PANAS–SF; [92,93])

This scale measures positive and negative affect and assesses the participants’ current mood state. It includes five adjectives covering positive mood (e.g., "excited," "inspired") and five adjectives covering negative mood states (e.g., "nervous," "guilty"). Participants are asked to show to what extent, at the time of the response, they feel the emotion represented in each item on a 5-point Likert scale ranging from 1, *"Very little or nothing"*, to 5 "*Extremely*". This instrument has good internal reliability (Cronbach’s α = 0.81 for positive affect, 0.88 for negative; [93]).

#### Game-related questionnaire

Participants were asked an open question regarding their strategy during the game. They were also asked to indicate to what extent they were close to the other participants and how much they cooperated (with the other participants present in the game) in their daily life on a 7-point Likert scale, from 1, *"Very little or nothing"* to 7, "*Extremely*". Also, participants were asked four questions regarding the execution of the task: (a) how pleasant it was; (b) how stressful it was; (c) how difficult it was; and (d) how well they understood both the instructions and the task on a 7-point Likert scale, from 1, *"Very little or nothing"* to 7, "*Extremely*".

### Procedure

The study was conducted in the behavioral lab of the School of Psychology at the University of Minho. Participants arrived at the lab in groups of four and were randomly assigned to one condition. More specifically, participants were divided into four conditions in a 2 (game type: Deterministic Prisoner’s Dilemma [DPD] vs. Stochastic Prisoner’s Dilemma [SPD]) × 2 (decision time: time delay vs. time constraint) between-subject design. Each participant used one computer to make their decisions. Computers were placed in the experimental room and in separate stations enclosed by cubicle dividers to provide anonymity. Participants were approximately 150 cm apart from each other. The informed consent was presented first, followed by the sociodemographic questionnaire and the Positive and Negative Affect Schedule (PANAS-SF) for all participants, then participants played the game task. At the end of the game task, they answered the game-related questionnaire, the PANAS-SF, and completed the Balloon Analogue Risk Task (BART). Finally, participants answered the Big Five Inventory, and the Interpersonal Reactivity Index (IRI) presented in a counterbalanced order.

During the game phase, initially, participants received instructions regarding the structure and rules of the game on the computer screen. The structure of the game participants learned differed according to the condition they were attending. On the SPD, the outcomes of alternative strategies were uncertain (see Table 1). Each player knew that cooperation (i.e., to invest in a risk-reducing measure) without the corresponding cooperation from their opponent (the participant present in the group they played against) would define probabilities of losses. By removing the uncertainty and replacing the values and probabilities in the SPD with the expected values (value x the corresponding probability) (see Table 2), the corresponding DPD was obtained (see Table 3). The values used in the two games were the same as in a previous study [44].

**Table 2:** Possible outcomes in the stochastic prisoner’s dilemma game.

	Player 2		
Player 1		Invest	Not Invest
	Invest	-45; -45	20% lose 145, 80% lose 45; 40% lose 100, 60% lose 0
	Not Invest	40% lose 100, 60% lose 0; 20% lose 145, 80% lose 45	52% lose 100, 48% lose 0; 52% lose 100, 48% lose 0

**Table 3:** Possible outcomes in the deterministic prisoner’s dilemma game.

	Player 2		
Player 1		Invest	Not Invest
	Invest	-45; -45	-65; -40
	Not Invest	-40; -65	-52; -52

Consistent with previous research, we called each sequence of ten rounds played with the same partner a supergame [44]. Participants played ten supergames of ten rounds each, totaling 100 decisions (the number of supergames was not disclosed to prevent end effects). Participants played against the same player in each supergame and then randomly switched players in the next supergame. At the start of each supergame, each player was given 1500 points, which they used in the game to decide if they wanted to invest or not. The game was described in a loss frame where participants incur a cost to make an investment decision. Each participant decided whether to invest the initial 1500 points to avoid a more significant financial loss.

Before initiating the game task, participants received instructions regarding their experimental condition–time delay or time pressure [61]. In the time pressure condition, participants were asked to make each decision as quickly as possible and were told they could not take longer than 10 seconds. In this condition, a timer appears on the screen to show the time left to decide, and in the last 3 seconds, the timer number is displayed in red. Still, participants could respond after 10 seconds, and the clock showed a warning that the time had ended. On the other hand, in the time delay condition, participants were asked to consider their decision carefully and were asked not to decide for at least 10 seconds (no timer appears, and they could choose at any moment).

**Fig 1:**
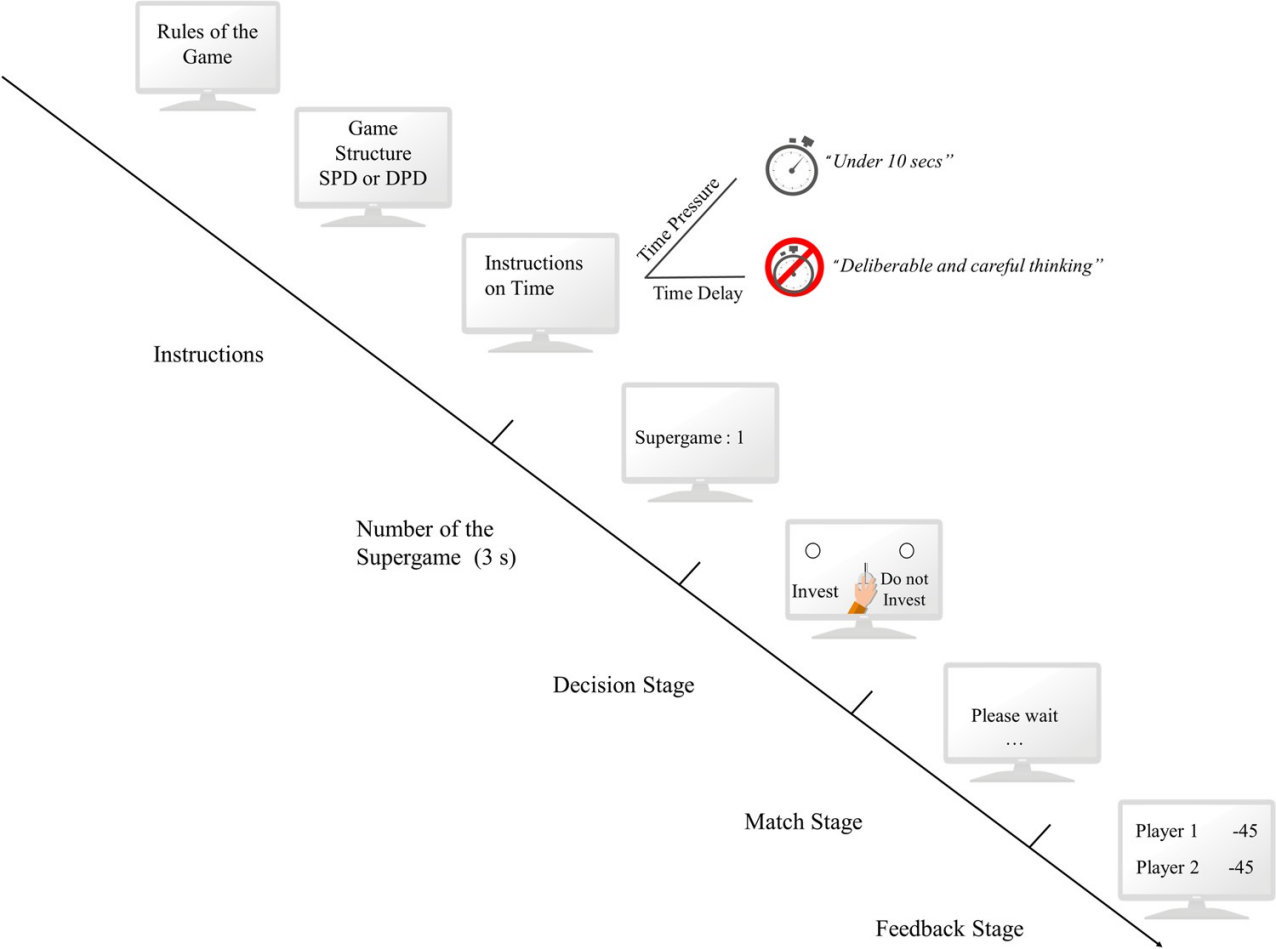
Representation of the game task.

Then, participants see the number of the super game they would play during 3 seconds on the screen. When the decision screen appeared, participants saw the game structure (congruent with the condition they were in) and the following instructions: "*The other player is also making a decision now*. *You will not know their decision until you submit your decision*. *So please make your investment decision now and submit it*." Participants then could decide whether they wanted to invest or not–*decision stage*. After making a decision, participants were asked to wait while the other player was deciding–designated by the *match stage*. After both participants made their decisions, they received information on both decisions and the payoffs–*feedback stage*. Then, a new decision stage would start. For a representation of the procedure, see Fig 1. In the time pressure condition, the total task had an approximate duration of 10 minutes, whereas it lasted for approximately 25 minutes in the time delay condition.

The entire session’s duration (including all questionnaires) was approximately 45 minutes, depending on the experimental condition participants were assigned. The BART was programmed in E-Prime (Version 2.0) [94], whereas the game was developed using z-Tree version 4.1.11[95], a software for economic experiments.

## Data analysis

We first assess the effects of uncertainty and time manipulation on participants’ likelihood to cooperate in the repeated Prisoner’s Dilemma games by conducting four logistic regressions in R (version 3.6.3;[96]).

The logistic regression model analyzed how investment decisions depended on *decision time* and the *type of games* and examined the interaction between uncertainty and *decision time*. In our study, participants made a dichotomic choice (Invest or Not invest) in each round, and throughout the session, each participant made a total of 100 decisions. We applied the same analysis as previous studies [44], a generalized mixed-effect logistic regression model. Two fixed effect variables were included to control the round and supergame order effect, one for round and one for supergame. In addition, a random effect variable (α) was included in the logistic regression to address interdependency among repeated observations, as shown in Eq (1). Specifically, we regressed each decision (1 for Investing, 0 for Not Investing) as the dependent variable on the following independent variables: *type of game* (DPD vs.SPD), *decision time* (time pressure vs. time delay), the interaction of *decision time* and *game type*, round numbers and supergame numbers. The generalized mixed-effect logit model used in this analysis can be written as:

$$\log(\frac{p_{ijk}}{1-p_{ijk}})=\beta_{0}+\beta_{1}{Type\;of\;Game}_{ijk}+\beta_{2}Decision{Time}_{ijk}+\beta_{3}{Type\;of\;Game}_{ijk}{DecisionTime}_{ijk}+\beta_{4}{SuperGame}_{j}+\beta_{5}{Round}_{k}+\alpha_{i}+\varepsilon_{ijk}$$ (1)

Eq (1) was estimated using the lme4 package in R [44,97].

Additionally, we analyze participants’ reaction time across the conditions and the rounds played. Following previous research [98], reaction times were log10 transformed to account for a heavily skewed distribution. Finally, we explore whether there were any individual differences across the four conditions. In the Supporting Information files, we included the results and discussion of further analyses exploring the role of individual differences in explaining the average individual cooperation rate (see S1 Appendix).

## Results

### Logistic regression models for the likelihood of cooperation in the first 50 rounds played

**Fig 2:**
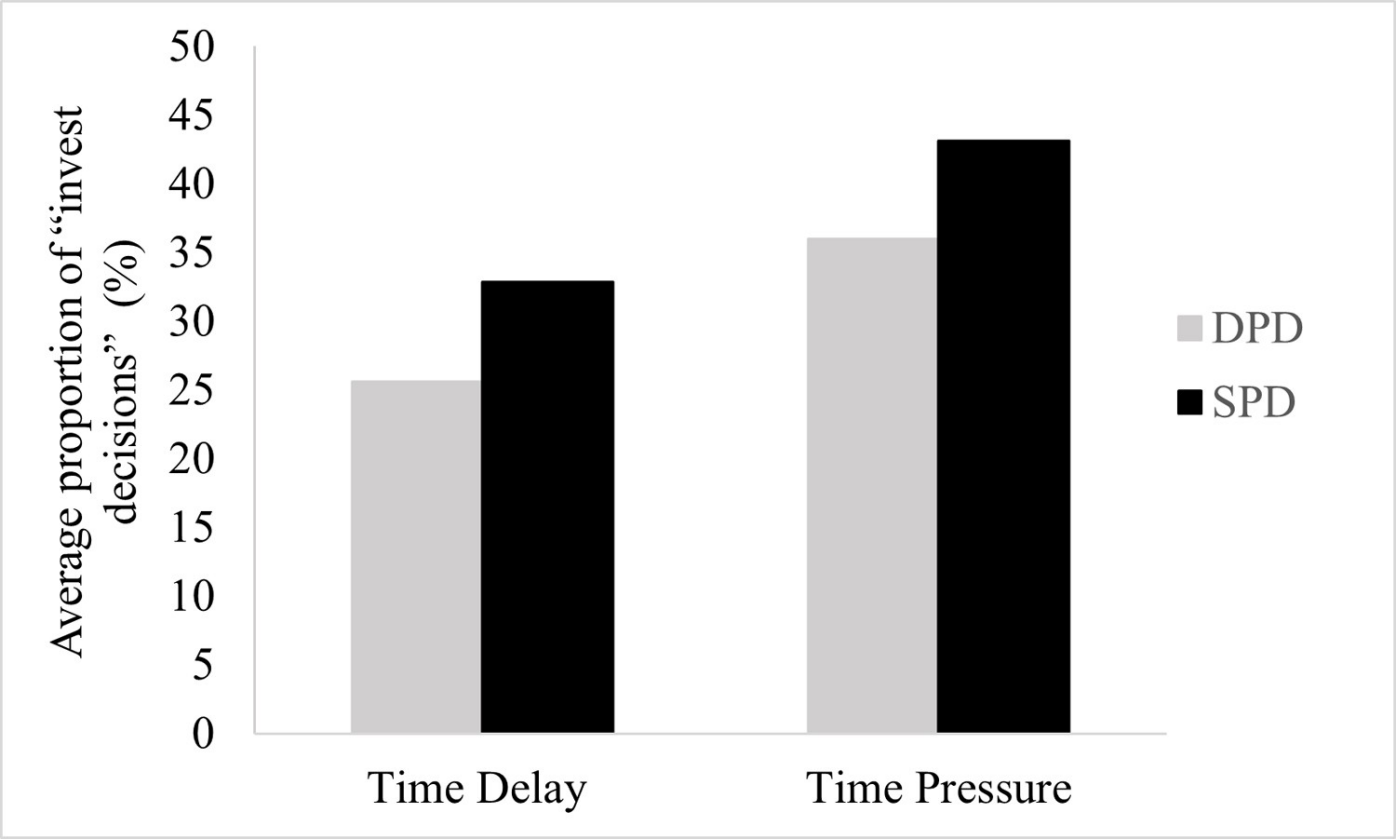
Cooperation rates in the four conditions across the first 50 decisions participants made. *n* = 28 participants in each condition presented. Error bars indicate standard errors of the mean.

In order to assess the effects of uncertainty and time manipulation on participants’ likelihood to cooperate in the repeated Prisoner’s Dilemma games, we start by analyzing the first 50 decisions made. Fig 2 shows the average cooperation rate (the proportion of times players invested) from the first 50 decisions. Participants in the time pressure condition appear to cooperate more than the time delay condition. Cooperation rates appear higher in the stochastic prisoner’s dilemma in both decision time manipulation.

The model applied revealed a significant effect of time pressure manipulation (*β*_2_ = .75, *z =* 2.07, *p =* .04, *OR =* 2.11, 95% CI = 1.04, 4.28), such that participants were more likely to cooperate under time pressure. However, there was not a significant effect in the type of game played (*β*_1_ = .51, *z* = 1.42, *p* = .16, *OR* = 1.67, 95% CI = .82, 3.39) or an effect of interaction,(*β*_3_ = .19, *z* = -.38, *ρ* = .70, *OR* = .82, 95% CI = .30, 2.22). However, the data showed an interesting effect as participants played the game repeatedly: the number of investment decisions decreased significantly as the sequence of supergames advanced (*β*_4_ = -.08, *z* = -3.52, *p*<.001, *OR* = .92, 95% CI = .88, .97) and with the advance of round within each supergame (*β*_5_ = -.08, *z* = -7.49, *p* < .001, *OR* = .92, 95% CI = .90, .94) (see Table 4).

**Table 4:** Estimates of the logistic regression model for cooperation probability in the first 50 rounds played.

	*β (SE)*	*z value*	*p*	95% CI for odds ratio		
				Lower	Odds Ratio	Upper
Intercept	-.71(.28)					
Type of Game	.52(.36)	1.42	.157	.82	1.67	3.41
Time manipulation	.75(.36)	2.07	.038*	1.04	2.12	4.33
Round2	-.47(.14)	-3.35	< .001	.48	.63	.82
Round3	-.44(.14)	-3.14	.002	.49	.65	.85
Round4	-.60(.14)	-4.27	< .001	.42	.55	.72
Round5	-.64(.14)	-4.56	< .001	.40	.53	.69
Round 6	-.55(.14)	-3.91	< .001	.44	.58	.76
Round 7	-.68(.14)	-4.84	< .001	.38	.51	.67
Round 8	-.87(.14)	-6.12	< .001	.32	.43	.55
Round 9	-.89(.14)	-6.20	< .001	.31	.41	.55
Round 10	-.94(.14)	-6.54	< .001	.29	.39	.52
Supergame2	-.01(.10)	-.13	.900	.81	.99	1.20
Supergame3	-.22(.10)	-2.11	.027	.65	.80	.97
Supergame4	-.17(.10)	-1.65	.098	.69	.85	1.03
Supergame5	-.32(.10)	-3.14	.002	.60	.73	.89
Game type x Time manipulation	-.20(.51)	-.40	.689	.30	.81	2.22
AIC	6088.4					
Log-likelihood	-3026.2					
Number of obs.	5600					

Note: AIC- The Akaike information criterion; CI- Confidence Interval; SE- Standard Error of the coefficient; *β*–Coefficient.

*** *p* < .001, ** *p* < .01

* *p* < .05.

### Logistic regression models for the likelihood of cooperation in the last 50 rounds played

To compare with the results from the first 50 decisions we applied a mixed-effects logistic regression model to the last 50 decisions participants made. The model demonstrated no significant effect on the type of game played (β₁ = -.19, z = -.38, p = .71, OR = .83, 95% CI = .30, 2.5). Additionally, there was no significant effect of the time manipulation (β₂ = -.03, z = -05, p = .96, OR = .98, 95% CI = .35, 2.68), and there was no interaction effect between the type of game played and the time manipulation (β₃ = .68, z = .95, p = .34, OR = 1.98, 95% CI = .48, 8.17).

However, the data showed a marginal significant effect as participants played the game repeatedly in supergames (β₄ = -.05, z = -1.92, p = .056, OR = .95, 95% CI = .91, 1.00) the number of investment decisions decreased as the sequence of supergames advanced. Within each supergame, there is a significant effect of the ten rounds played (β₅ = -.11, z = -9.10, p < .001, OR = .89, 95% CI = .87, .92), implying that participants learned not to cooperate over time within the supergame (for more details see Table 5).

**Table 5:** Estimates of the logistic regression model for cooperation probability in the last 50 rounds played.

	*β (SE)*	*z value*	*p*	95% CI for odds ratio		
				Lower	Odds Ratio	Upper
Intercept	-.85(.36)	-2.21	.027			
Type of Game	-.18(.51)	-.36	.720	.30	.83	2.28
Time manipulation	-.03(.52)	-.05	.956	.35	.97	2.69
Round2	-.24(.15)	-1.68	.093	.58	.78	1.04
Round3	-.45(.15)	-2.99	.003	.48	.64	.86
Round4	-.46(.15)	-3.07	.002	.47	.63	.85
Round5	-.43(.15)	-2.90	.004	.48	.65	.87
Round 6	-.61(.15)	-4.00	< .001	.41	.54	.73
Round 7	-.60(.14)	-3.99	< .001	.41	.55	.74
Round 8	-.89(.14)	-5.72	< .001	.30	.41	.56
Round 9	-1.03(.16)	-6.52	< .001	.26	.36	.49
Round 10	-1.15 (.16)	-7.18	< .001	.23	.31	.43
Supergame 7	.37(.11)	3.36	.001	1.17	1.44	1.79
Supergame 8	.06(.11)	.50	.619	.85	1.06	1.31
Supergame 9	.11(.11)	.96	.336	.90	1.11	1.38
Supergame 10	-.10(.11)	-.88	.377	.73	.91	1.13
Game type x Time manipulation	.68(.73)	.94	.346	.48	1.98	8.24
AIC	5286.0					
Log-likelihood	-2625.0					
Number of obs.	5600					

Note: AIC- The Akaike information criterion; CI- Confidence Interval; SE- Standard Error of the coefficient; β–Coefficient.

*** p < .001, ** p < .01, * p < .05.

### Logistic regression models for the likelihood of cooperation in the 100 rounds played

**Fig 3:**
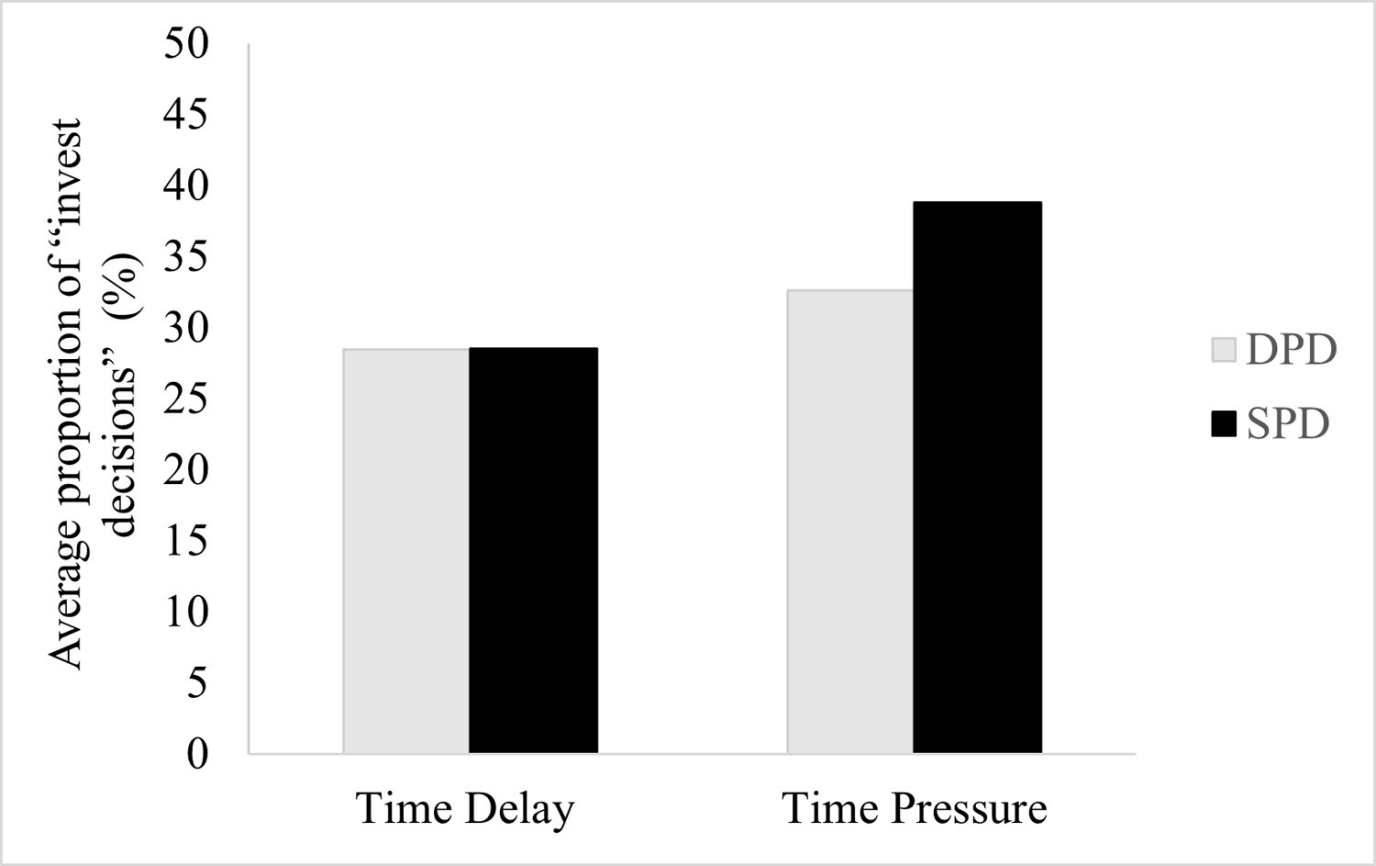
Cooperation rate between the manipulation of time pressure and the type of game across all 100 decisions. *n* = 28 participants in each condition presented. Error bars indicate standard errors of the mean.

The average cooperation rate (the proportion of times players decided to invest) from the 100 decisions participants made is shown in Fig 3. Fig 3 suggests that participants under time pressure manipulation were more cooperative than under time delay manipulation. Participants in the time pressure condition and who played the SPD game have a higher average proportion of cooperation (M = 0.39) than the other three conditions. Also, participants in the time-delay condition have a similar average proportion of cooperative responses in the DPD (M = 0.28) and the SPD (M = 0.29).

**Fig 4:**
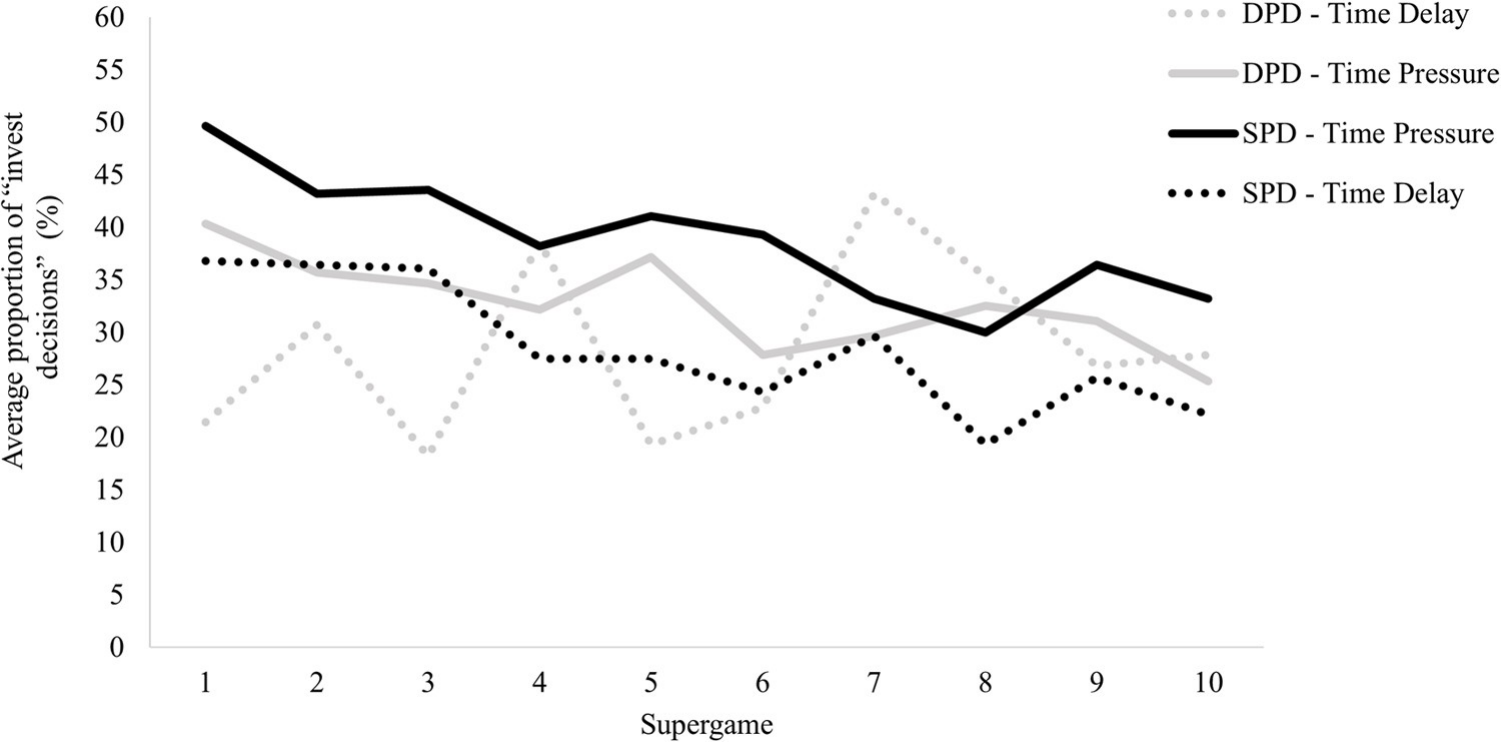
Cooperation rates across the ten supergames played in the four conditions. *n* = 28 participants in each condition presented. A version of this figure with error bars is presented in the supporting information (S1 Fig).

To confirm these differences, we applied a mixed-effects logistic regression model to a total of 100 decisions participants made. The model demonstrated no significant effect on the type of game played (*β*_1_ = .22, *z* = .57, *p =* .57, *OR* = 1.24, 95% CI = .58, 2.66). Additionally, there was no significant effect of the time manipulation (*β*_2_ = .47, *z* = 1.22, *p* = .22, *OR* = 1.60, 95% CI = .75, 3.43), and there was no interaction effect between the type of game played and the time manipulation (*β*_3_ = .06, *z* = .12, *p =* .*91*, *OR = 1*.*07*, 95% CI = .37, 3.11). The supergame (*β*₄ = -.05, *z* = -6.87, *p <* .*001*, *OR =* .*95*, 95% CI = .93, .96) and round (*β*₅ = -.09, *z* = -11.32, *p <* .*001*, *OR =* .*91*, 95% CI = .90, .93), appeared to follow the same pattern as in the prior model tested. The number of investment decisions decreased significantly as the sequence of supergames advanced and with the advance of round within each supergame implying that participants learned not to cooperate over time (for more details see Table 6 and the graphic representention of this tendency in Fig 4).

**Table 6:** Estimates of the logistic regression model for cooperation probability.

	*β (SE)*	*z value*	*p*	95% CI for odds ratio		
				Lower	Odds Ratio	Upper
Intercept	-.53 (.29)					
Type of Game	.22 (.39)	.57	.57	.58	1.25	2.67
Time manipulation	.47 (.39)	1.22	.22	.75	1.61	3.44
Round2	-.35 (.10)	-3.51	< .001	.58	.71	.86
Round3	-.42 (.10)	-4.20	< .001	.54	.66	.80
Round4	-.51 (.10)	-5.07	< .001	.50	.61	.73
Round5	-.52 (.10)	-5.17	< .001	.49	.60	.73
Round6	-.54(.10)	-5.42	< .001	.48	.58	.71
Round7	-.61(.10)	-6.09	< .001	.45	.54	.66
Round8	-.83(.10)	-8.14	< .001	.36	.43	.53
Round9	-.90(.10)	-8.71	< .001	.33	.41	.50
Round10	-.98(.10)	-9.38	< .001	.31	.38	.46
Supergame2	-.02(.10)	-.15	.88	.81	.99	1.20
Supergame3	-.23(.10)	-2.33	.03	.65	.80	.97
Supergame4	-.17(.10)	-1.67	.09	.69	.85	1.03
Supergame5	-.32(.10)	-3.17	.002	.59	.72	.88
Supergame6	-.49(.10)	-4.73	< .001	.50	.61	.75
Supergame7	-.16(.10)	-1.63	.104	.70	.85	1.03
Supergame8	-.44(.10)	-4.31	< .001	.53	.64	.79
Supergame9	-.39(.10)	-3.89	< .001	.55	.67	.82
Supergame10	-.58(.10)	-5.58	< .001	.46	.56	.69
Game type x Time manipulation	.06(.55)	.12	.91	.36	1.07	3.11
AIC	11636.6					
Log-likelihood	-5795.3					
Number of obs.	11200					

Note: AIC- The Akaike information criterion; CI- Confidence Interval; SE- Standard Error of the coefficient; *β*–Coefficient.

*** *p* < .001, ** *p* < .01, * *p* < .05.

**Fig 5:**
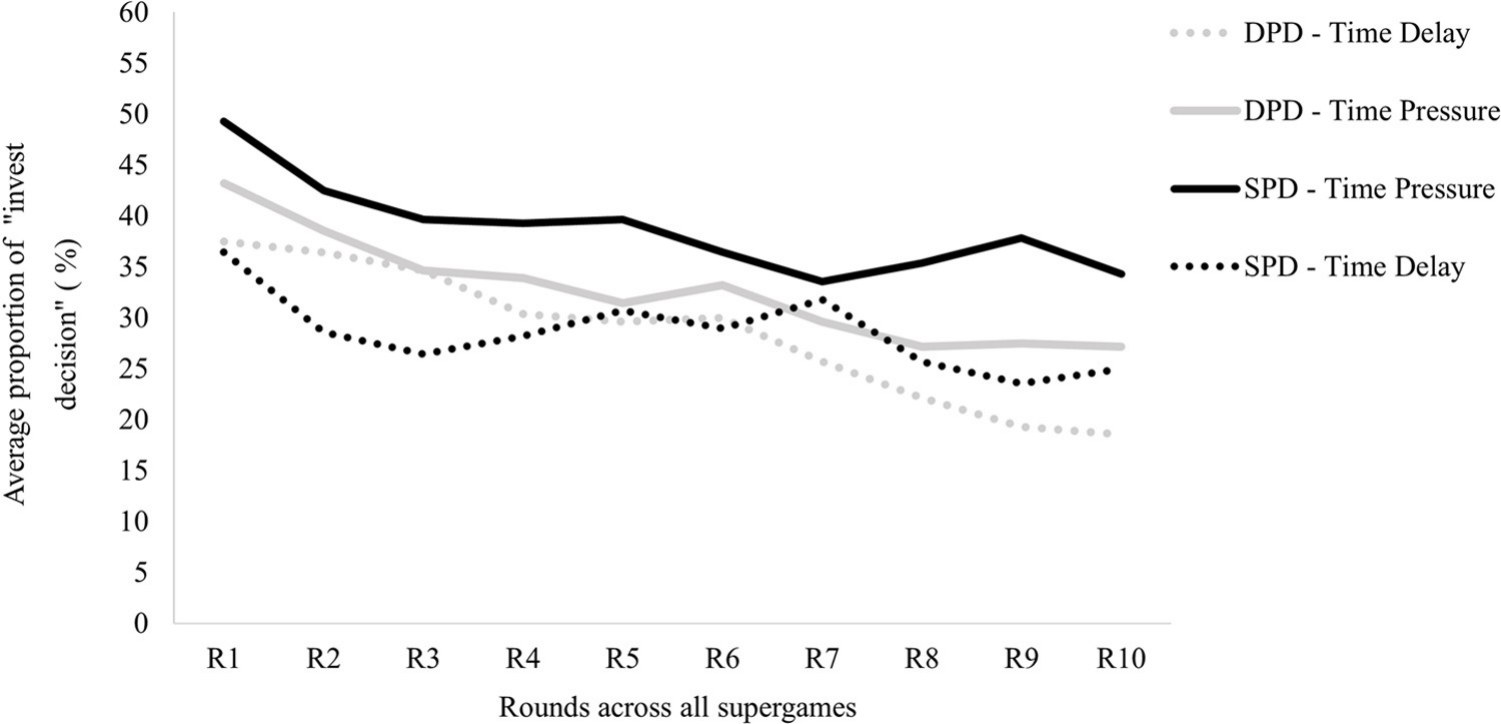
Mean cooperation rates across the rounds played in all supergames in the four conditions. *n* = 28 participants in each condition presented. A version of this figure with error bars is presented in the supporting information (S2 Fig).

Additionally, we apply a mixed-effect logistic regression to further explain the decreasing cooperation results across the rounds and the supergame. Specifically, we regressed each decision (1 for Investing, 0 for Not Investing) as the dependent variable on the following independent variables: supergame number, round number, and the interaction of supergame and round. The model revealed a significant effect of the round number (*β* = -.050, z = -2.90, *p* = .004, *OR* = .95, 95% CI = .92, .98) and a significant effect of interaction between the supergame and the round (*β* = -.008, z = -2.78, *p* = .005, *OR* = .99, 95% CI = .99, 1.00), such that participants learned not to cooperate over the increasing of rounds but cooperate in beginning of each supergame (see Fig 5 for more details). However, there was not a significant difference in the supergame played (*β* = -.014, z = -2.78, *p* = .412, *OR* = .99, 95% CI = .95, 1.02).

### Reaction time analysis

**Fig 6:**
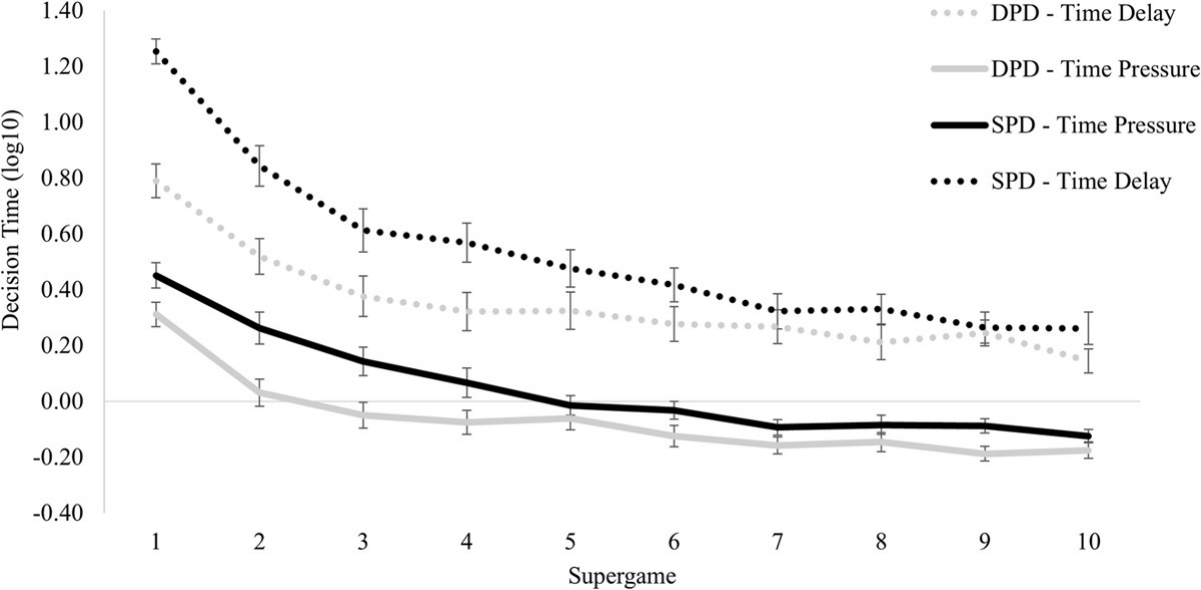
The average amount of decision time across conditions and rounds played. *n* = 28 participants in each condition presented. Error bars indicate standard errors of the mean.

Finally, we examined the impact of ’participants’ average decision time in log 10 transformation on the different rounds played for the four conditions (Fig 6).

A 4 (between-subjects factor: DPD-Time Delay versus DPD- Time Pressure versus SPD- Time Delay versus SPD- Time Pressure) by 10 (within-subjects factor: supergames) by 10 (within-subjects factor: rounds) mixed design ANOVA was conducted on the average participant’s decision time. We used this model to examine the effect of the different conditions controlling for the different individual reaction times in each round participants play.

The main effect of the condition was statistically significant, *F*(3, 108) = 41.62, *p* ≤ .001, *η*^2^ = .536; Pairwise comparisons reveal that participants under time pressure conditions in both types of games have faster reaction times compared to the time delay condition. The two types of games in time pressure manipulation do not differ statistically between them *p* = .064 (Mean DPD = -0.06; Mean SPD = 0.05). Participants in the SPD-Time delay condition reveal slower reaction times (Mean = 0.53), followed by participants on the DPD-Time delay condition (Mean = 0.35, see Fig 6).

**Fig 7:**
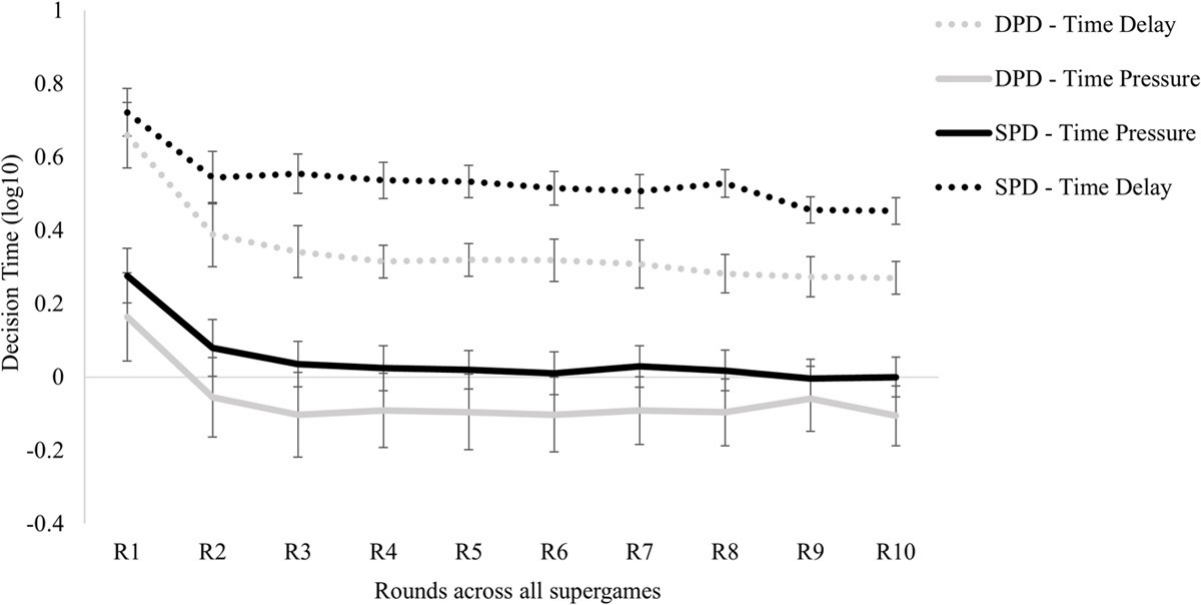
The average amount of decision time across the rounds played in all supergames. *n* = 28 participants in each condition presented. Error bars indicate standard errors of the mean.

The main effect of the supergame was significant, *F*(9,972) = 180.327, *p* < .001, *η*^2^ = .625, meaning that as the experiment progressed, the reaction time decreased, and also the main effect of the rounds *F*(9,972) = 98.419, *p* < .001, *η*^2^ = .477, revealing that as the rounds augment the reaction time diminish. The interaction between these two repeated measure variables were also significant *F*(81,8748) = 2.972, *p* < .001, *η*^2^ = .027. With the increase of the game time (supergame x round), participants’ reaction time diminishes (see Fig 7). Likewise, the two-way interactions between supergame and condition (F(27,972) = 6.280, p < .001, *η*^2^ = .149) and round and condition (*F*(27,972) = 2.414, p < .001, *η*^2^ = .063) were also statistically significant. Besides the interaction effect between the supergame, the round and the condition were not significant *F*(243,8748) = 1.116, *p* = .106, *η*^2^ = .030.

In order to explore whether there were any differences across the four conditions regarding individual variables we conducted an ANOVA. Results reveal that there were no differences across the four conditions regarding the individual risk-taking propensity (*p* = .145), personality factors (BFI_extraversion *p* = .358; BFI_agreeableness *p* = .452; BFI_conscientiousness *p* = .727; BFI_neuroticism *p* = .138; BFI_openess *p* = .321), empathy (TP *p* = .520; PE *p* = .504; DP *p* = .117; F *p* = .388), positive and negative affect (Pos. affect *p* = .306;Neg. Affect *p* = .468; End_Pos. Affect *p* = .538; End_Neg. Affect *p =* .*340*). Additionally Kruskal-Wallis test were apply to analyse whether there were any differences across the four conditions regardind the sociodemographic variables and the questions regardind the game related questionnaire. Resuls reveal that there were no diferences across the sociodemographic variables(Age *p* = .340; Gender *p* = .936; SES *p* = .610), and game-related questionnaire (Cooperate *p* = .713; Proximity *p* = .162, Pleasant *p* = .473; Difficulty experienced *p* = .097).

## Discussion

The main goal of this study was to analyze the simultaneous impact of time pressure and uncertainty on cooperation in a prisoner’s dilemma game under uncertainty. We observed participants’ decisions in repeated interactions for ten rounds, then another ten rounds played against another randomly chosen partner, and so on, for ten sets of rounds (the supergames). Participants were informed of these aspects but not of the total number of supergames they were expected to play. In a repeated PD game, we manipulated cognitive processes using time pressure manipulation methods on their decision. Under a time pressure condition, participants are asked to decide as quickly as possible (< 10 s). In a time delay condition, participants were asked to consider their decision carefully. Finally, environmental uncertainty was manipulated by having PD games with defined versus probabilistic outcomes. Studying how participants behave in such environments helps us learn about the determinants of cooperation.

Consistent with our first hypothesis and findings from other studies [4,44,46], the data revealed a learning process as participants played the game repeatedly. Results showed that as the number of rounds and supergames increased, individuals learned not to cooperate with others. Even though several studies in the field of prisoner’s dilemma have been conducted over the years, research on the area still tries to understand whether people learn to cooperate or defect in the game [99,100]. Different studies have given contradictory results of the evolution of play with experience [100]. A recent study tried to understand cooperative behavior and its evolution with experience, and results reveal that the parameters of the supergame have a significant impact on initial cooperation rates [100]. For example, the longer the extension of the supergame, the greater the initial cooperation rates are because conditionally cooperative strategies increase their value [100]. The authors identify the interaction of two opposing processes–learning to cooperate in early rounds by convergence to using threshold strategies and learning to defect in later rounds due to the argument of backward induction–to be fundamental in explaining the variation across papers and treatments in the evolution of behavior [100]. Interestingly, our data show that cooperation rates increase at the beginning of each new supergame (a new interaction) but a little less at each further interaction.

Regarding the effect of intuition on cooperation, results are consistent with previous studies [81] and predictions of the Social Heuristic Hypothesis [62,68]. Likewise, the results of applying the logistic regression model to the first fifty rounds are consistent with the second hypothesis, revealing that individuals tend to rely on a more intuitive decision-making process to cooperate. In addition, these results were confirmed in previous findings, where individuals appear to be more inclined to cooperate with others under time pressure than under time delay [62]. However, these findings conflict with previous studies that found that deliberation plays an essential role in cooperation [75].

The second and third logistic regression models did not reveal an effect of time pressure manipulation on cooperation. This result is consistent with our third hypothesis that intuition would not affect participants’ decisions as the game progress. Furthermore, based on the SHH predictions, intuition was expected not to affect games where it could be payoff-maximizing to cooperate (e.g., games in which reciprocity was possible) [54,62,68,101]. However, other studies exploring the effects of intuition on cooperation did not find an effect of time pressure manipulation on cooperation even in one-shot games [5,74,79].

Our data appears to support the idea that the cooperative default response varies with the absolute level of cooperation a person faces in the game. We found that participants learn not to cooperate with others as the game progresses. As predicted by the SHH in a repeated game, intuition does not affect cooperation. On the contrary, time pressure manipulation favors cooperation in the first five supergames. The average cooperation rate is higher in the first decisions participants made than the average cooperation rate of the last decisions.

Similar to previous research, our results also demonstrate the importance of considering the social environment when examining decision time [81]. This explanation of the effect of the social environment on decision time can explain why there are mixed results in the literature and why results vary with the social environment within repeated game studies [81,85]. Other studies also concluded that cooperation is a learning process from their study where they compared experienced participants in playing social dilemmas games versus inexperienced participants [69]. The study revealed that experienced participants in this task under time pressure manipulation were significantly more cooperative than naive participants. This effect appears to be even more surprising considering that other studies report that experience negatively affects cooperation among residents in the US [63]. Both studies are in line with SHH, which assumes that experience operates mainly through intuition, but it does not predict the sign of the effect of experience. Furthermore, the SHH posits that individuals who experience cooperation in their daily lives will lead toward cooperation as the intuitive response, but not those who live in a non-cooperative setting [63,69,81].

It is worth noting that there are four participants present in the room playing the game, and participants play simultaneously one supergame with one partner and then switch to another. Although it is random whom they play along in the supergame, it appears that when they repeat the interaction they previously had, time pressure manipulation does not have an effect, and non-cooperative behavior is augmented. Cooperation was higher than in the other rounds at the beginning of each new supergame (new interaction). This finding is consistent with the idea that daily experiences with norms and institutions initially led participants to expect others to cooperate and be inclined towards cooperation themselves. However, once participants start the game and learn about the behavior of their partners, they follow cues from the social environment. This result is consistent with the Social Heurist Hypothesis findings that emphasize that experiences from outside the lab influence participants’ decisions. A recent study using a different statistical model–the Drift Diffusion Model- also found that people’s initial intuitive decision is to cooperate [102]. However, deliberation becomes dominant over an initial intuitive bias towards cooperation if the game is repeated. When we play a game where most players are defectors, the intuitive decision progressively becomes to defect. In contrast, when we play with cooperators, we become even more biased towards cooperation. Similar to our findings, the authors also found that this initial cooperation tendency is resilient, as, after a short pause, it resets to the same initial value [102].

One more interesting result from observing participants’ decision time and cooperation rates reveal that time pressure manipulation decreases the rate of participants changing their strategy. Another possible explanation for the positive result between time pressure manipulation and cooperation in the first 50 decisions made but not in the total decisions is that extreme time pressure decreases cooperative behavior [74,98]. Analyzing the reaction time, we can see that participants in the analysis of the 100 decisions were quicker than in the beginning fifty decisions. As long as the game progressed, participants made their decisions quickly. Our results appear to support the idea of an inverted-U relationship between reaction time and cooperation found in the literature where extreme time pressure manipulation decreases cooperation. In contrast, light time pressure increases cooperation [74]. In our results, the effect of time pressure manipulation is only significant on the first fifty decisions, where reaction times were higher than in the analysis of all decisions.

### Effects of uncertainty on cooperation

Our data did not confirm our fourth hypothesis regarding the game’s primary effect of uncertainty on cooperation. More specifically, we did not find an effect of the type of game (when uncertainty is present or not) on cooperation. These results are inconsistent with previous studies [18,28,44]. The average cooperative response across conditions suggests that participants under environmental uncertainty were more cooperative than participants who played the deterministic form of the game. Previous research found that social uncertainty undermines cooperation when people believe their choice is critical (significantly impacting the outcome) [103]. When criticality is low, there was a slight increase in cooperation rates with uncertainty. Based on this study, we can also suppose that participants do not perceive their choice as critical, especially in a stochastic game version where the results are based on probabilistic outcomes.

Recent debates stated that uncertainty does not affect uniformly social interactions [34,104]. Recently, a study revealed that uncertainty does not always promote selfish behaviors [105]. Instead, they found that an individual’s prosocial behavior was increased under impact uncertainty (uncertainty about how the negative outcome will impact others’ well-being). The authors claim that individuals are more selfish when they are uncertain about what outcomes their decisions will produce for others but less selfish when they are uncertain about the impact of those outcomes on others’ welfare [105].

Some studies have been interested in analyzing whether and when participants play cooperatively and the strategies used in a repeated prisoner’s dilemma with uncertainty [83]. The study revealed that individuals tend to cooperate in repeated games with uncertainty when there is a cooperative equilibrium (in which both players start by cooperating and continue to do so until the first person defect). The authors report that strategies, such as TF2T (individual cooperates unless the other player chose to defect in both of the last two rounds) that involves forgiveness is often used and can successfully obtain high payoffs given the actual distribution of play. However, it is not an equilibrium for all individuals to play TF2T. The authors concluded that forgiving others and cooperating in an uncertain world could be payoff-maximizing than quickly reciprocating and defect [83]. Therefore, it would be necessary also to analyze the strategies used in the two games we present and understand which game strategy is a good predictor of cooperation even in a prisoner’s dilemma game with probabilistic outcomes.

The results found regarding uncertainty can also be explained, considering the game structure’s methodological option. The results found regarding uncertainty can also be explained, considering the game structure’s methodological option. For example, in the repeated version of the Prisoners Dilemma game is defined that the game structure should follow the following rule: *T > R > P > S*; however, it is often required that *R < (S + T)/2* so that the continuous cooperation is better than alternating between cooperation and defection [21,99]. The games developed by previous research [44] and used in this study only follow the first rule. It is also important to note that it appears that participants, as the number of rounds, increased tens to reach for Nash equilibrium for iterated prisoner’s dilemma that is non-cooperation (*P*, *P*).

We did not find any interaction between the type of game and time pressure manipulation. Earlier, we hypothesized that time pressure manipulation augmented cooperative behavior under conditions of uncertainty. On the contrary, we expected that cooperative behavior would decrease in uncertain conditions under time delay. This study is one of the first to analyze environmental uncertainty and time pressure manipulation to the best of our knowledge. Analyzing mean data, participants in SPD conditions under time pressure have a higher average cooperate rate. Furthermore, the effect in the stochastic game augments with time pressure manipulation. However, the average cooperation rate is similar under time delay in both types of games.

### Limitations and further research

One crucial methodological option we should consider is that the game is in a loss frame. Therefore, we should consider the possibility that individual risk-seeking behavior in the loss frame might change when the game is played in the gain frame. For example, the current study uses a prisoner dilemma game with negative outcomes to mimic a real-world scenario where players invest in reducing the risks of suffering a loss. Researchers have found that people encode losses and gains differently [106] and typically show greater sensitivity to loss than gain [107]. Thus, individuals will make riskier choices to avoid losses than they will to produce gains.

A critical limitation of this study is that we did not assess participants’ comprehension regarding the game structure. Thus, understanding the game remains a potentially problematic aspect of the dominant methodological design in this area [54,71]. Additionally, participants were instructed to think carefully in the time delay condition and not decide for at least 10 seconds. However, they can respond at any second. Therefore, it is essential that future research force participants’ responses after a specific time.

Finally, another question concerns the payments for attending the experiment. In the present study, the reward is not directly correlated with the participant’s decisions (i.e., paying a participation fee) or because a payment based on repeated trials decreases the effects [108]. The reward provided in our studies are credit courses, and we additionally attribute a money prize to two randomly chosen participants from all participants who completed the task. Since participants did not know the total of participants who completed the experiment, they only saw the number of participants present in their group. In this case, there is uncertainty regarding the group size of receiving the final prize. Research in group size uncertainty revealed a positive effect on cooperation [47–52].

### Conclusions

To summarize, our study helps to clarify the mixed-effects found in the literature regarding the effectiveness of cognitive processing manipulation on cooperation [54]. Overall, our analysis suggests that, compared to time delay, time pressure leads to greater cooperation on the first rounds of interaction. Our data support the idea that the intuitive cooperative default response varies with the absolute level of cooperation an individual faces in the game. The results point to a direction in which participants cooperate less with their counterparts as the number of rounds increases. In addition, it would be helpful to try to understand better how participants learn. The intuition effect found does not appear to be maintained throughout the experiment’s entire duration, and neither can we rule out the effect of learning. Although our study does not find significant developments regarding the interaction effect of intuition and uncertainty under a repeated game, the generality of the findings in this area remains to be explored.

Research regarding dual-process manipulation in the last eight years has led to a better understanding of the personal and environmental factors that shape cooperation and prosocial behavior [54,101]. Understanding when and why people cooperate in our society, replicating situations that we encounter in real life, like risk and uncertainty, is undoubtedly a fundamental topic for further research.

## Supporting information

S1 Fig Cooperation rates across the ten supergames played in the four conditions. *n* = 28 participants in each graphic presented. Error bars indicate standard errors of the mean.
(TIF)

S2 Fig Mean cooperation rates across the rounds played in all supergames in the four conditions. *n* = 28 participants in each graphic presented. Error bars indicate standard errors of the mean.
(TIF)

S1 Table Instructions for the main task. (DOCX)

S1 Appendix Supplementary notes. The effects of individual differences on cooperation.
(DOCX)

S1 File Database. (XLSX)

## References

[1] Rand DG, Nowak MA. Human cooperation. Trends in Cognitive Sciences. 2013; 17(8): 413–425. doi: 10.1016/j.tics.2013.06.003 23856025

[2] Fehr E, Fischbacher U. The nature of human altruism. Nature. 2003;425: 785–791. doi: 10.1038/nature02043 14574401

[3] Axelrod R. The Evolution of Cooperation. New York: Basic Books; 1984.

[4] Fehr E, Fischbacher U. Social norms and human cooperation. Trends in Cognitive Sciences. 2004; 8(4): 185–190. doi: 10.1016/j.tics.2004.02.007 15050515

[5] Alós-Ferrer C, Garagnani M. The cognitive foundations of cooperation. Journal of Economic Behavior and Organization. 2020;175: 71–85. doi: 10.1016/j.jebo.2020.04.019

[6] Nowak MA. Five rules for the evolution of cooperation. Science.2006; 314(5805): 1560–1563. doi: 10.1126/science.1133755 17158317 PMC3279745

[7] Nowak MA. Evolving cooperation. Journal of Theoretical Biology. 2012; 299: 1–8. doi: 10.1016/j.jtbi.2012.01.014 22281519

[8] Hamilton WD. The Genetical Evolution of Social Behaviour. Journal of Theoretical Biology. 1964; 7(1): 17–52. doi: 10.1016/0022-5193(64)90039-6 5875340

[9] Trivers RL. The evolution of reciprocal altruism. The Quarterly Journal of Biology. 1971;46(1): 35–57. doi: 10.1086/406755

[10] Nowak MA, Sigmund K. Evolution of indirect reciprocity by image scoring. Nature. 1998;393(6685): 573–577. doi: 10.1038/31225 9634232

[11] Nowak MA, Sigmund K. Evolution of indirect reciprocity. Nature. 2005; 437(7063): 1291–1298. doi: 10.1038/nature04131 16251955

[12] Nowak MA, May RM. Evolutionary games and spatial chaos. Nature. 1992;359(6398): 826–9. doi: 10.1038/359826a0

[13] Ohtsuki H, Hauert C, Lieberman E, Nowak MA. A simple rule for the evolution of cooperation on graphs and social networks. Nature. 2006;441(7092): 502–5. doi: 10.1038/nature04605 16724065 PMC2430087

[14] Traulsen A, Nowak MA. Evolution of cooperation by multilevel selection. Proceedings of the National Academy of Sciences. 2006;103(29): 10952–10955. doi: 10.1073/pnas.0602530103 16829575 PMC1544155

[15] West SA, Griffin AS, Gardner A. Evolutionary Explanations for Cooperation. Current Biology. 2007;17(16): 661–672. doi: 10.1016/j.cub.2007.06.004 17714660

[16] Dawes RM, Messick DM. Social dilemmas. International Journal of Psychology. 2003;5: 111–116. doi: 10.1080/002075900399402

[17] Kollock P. Social dilemmas: The anatomy of cooperation. Annual review of sociology. 1988;24(1): 183–214. doi: 10.1146/annurev.soc.24.1.183

[18] van Lange PAM, Joireman J, Parks CD, van Dijk E. The psychology of social dilemmas: A review. Organizational Behavior and Human Decision Processes. 2013;120(2): 125–141. doi: 10.1016/j.obhdp.2012.11.003

[19] Dawes RM. Social Dilemmas. Annual Review of Psychology.1980;31: 169–193. doi: 10.1146/annurev.ps.31.020180.001125

[20] Capraro V. A Model of Human Cooperation in Social Dilemmas. PLoS ONE. 2013;8(8): doi: 10.1371/journal.pone.0072427 24009679 PMC3756993

[21] Axelrod R, Hamilton WD. The evolution of cooperation. Science.1981; 211(4489): 1390–1396. doi: 10.1126/science.7466396 7466396

[22] Taylor M. Anarchy and cooperation. London: Wiley; 1976. Available from: http://lib.ugent.be/catalog/rug01:000419542.

[23] Taylor M. The Possibility of Cooperation. Cambridge University Press; 1987.

[24] Rapoport A, Guyer M. A Taxonomy of 2×2 games. General Systems. 1966;11:203–214.

[25] Gracia-Lázaro C, Cuesta JA, Sánchez A, Moreno Y. Human behavior in Prisoner’s Dilemma experiments suppresses network reciprocity. Scientific Reports. 2012;2: 1–4. doi: 10.1038/srep00325 22439103 PMC3309394

[26] Wang Z, Wang L, Yin ZY, Xia CY. Inferring reputation promotes the evolution of cooperation in spatial social dilemma games. PLoS ONE. 2012;7(7). doi: 10.1371/journal.pone.0040218 22808120 PMC3392274

[27] Rapoport A, Chammah AM. Prisoner’s dilemma: A study in conflict and cooperation. Ann Arbor: University of Michigan Press; 1965. doi: 10.3998/mpub.20269

[28] van Dijk E, Wit A, Wilke H, Budescu D V. What We Know (and Do Not Know) about the Effects of Uncertainty on Behavior in Social Dilemmas. In: Suleiman R, Budescu D V, Fischer I, Messick D M, editors. Contemporary psychological research on social dilemmas. New York, NY, US: Cambridge University Press; 2004. pp.315–331.

[29] Gangadharan L, Nemes V. Experimental analysis of risk and uncertainty in provisioning private and public goods. Economic Inquiry. 2009;47(1): 146–164. doi: 10.1111/j.1465-7295.2007.00118.x

[30] Vives ML, Feldmanhall O. Tolerance to ambiguous uncertainty predicts prosocial behavior. Nature Communications. 2018; 9(1): 2156–2165. doi: 10.1038/s41467-018-04631-9 29895948 PMC5997641

[31] Messick DM, Allison ST, Samuelson CD. Framing and Communication Effects on Group Members’ Responses to Environmental and Social Uncertainty. In Maital S, editor. Applied behavioral economics. New York: New York University Press; 1988. pp.677–700.Available from: https://www.researchgate.net/publication/246236173.

[32] Budescu D V, Rapoport A, Suleiman R. Resource dilemmas with environmental uncertainty and asymmetric players. European Journal of Social Psychology. 1990; 20(6): 475–487. doi: 10.1002/ejsp.2420200603

[33] Gustafsson M, Biel A, Gärling T. Overharvesting of resources of unknown size. Acta Psychologica. 1999; 103: 47–64. doi: 10.1016/S0001-6918(99)00024-4

[34] FeldmanHall O, Shenhav A. Resolving uncertainty in a social world. Nature Human Behaviour. 2019; 3(5): 426–435. doi: 10.1038/s41562-019-0590-x 31011164 PMC6594393

[35] Tversky A, Shafir E. The Disjunction Effect in Choice Under Uncertainty. Psychological Science. 1992; 3(5): 305–309. doi: 10.1111/j.1467-9280.1992.tb00678.x

[36] van Dijk E, Zeelenberg M. The Discounting of Ambiguous Information in Economic Decision Making. Journal of Behavioral Decision Making. 2003;16(5): 341–52. doi: 10.1002/bdm.450

[37] Jorgenson D 0, Papciak AS. The Effects of Communication, Resource Feedback, and Identifiability on Behavior in a Simulated Commons. Journal of Experimental Social Psychology. 1981;17(4): 373–385. doi: 10.1016/0022-1031(81)90044-5

[38] Sniezek JA, May R, Sawyer JE. Social Uncertainty and Interdependence: A Study of Resource Allocation Decisions in Groups. Organizational Behavior and Human Decision Processes. 1990;46(2): 155–180. doi: 10.1016/0749-5978(90)90027-7

[39] Wilke HAM, Braspenning J. Reciprocity: Choice shift in a social trap. European Journal of Social Psychology. 1989;19(4): 317–326. doi: 10.1002/ejsp.2420190406

[40] Budescu VD, Rapoport A, Ramzi S. Common Pool Resource Dilemmas under Uncertainty: Qualitative Tests of Equilibrium Solutions. Games and Economic Behavior. 1995;10(1): 171–201. doi: 10.1006/game.1995.1029

[41] Hine DW, Gifford R. Individual Restraint and Group Efficiency in Commons Dilemmas: The Effects of Two Types of Environmental Uncertainty’. Journal of Applied Social Psychology. 1996;26(11): 993–1009. doi: 10.1111/j.1559-1816.1996.tb01121.x

[42] Rapoport A, Budescu D v, Suleiman R, Weg E. Social dilemmas with uniformly distributed resources. In: Liebrand W, Messick D, Wilke H, editors. Social dilemmas: Theoretical issues and research findings. Elmsford, NY, US: Pergamon Press; 1992. pp. 43–57.

[43] Suleiman R, Budescu D V. Common Pool Resource (CPR) dilemmas with incomplete information. In: Budescu D V, Erev I, Zwick R, editors. Games and human behavior: Essays in Honor of Amnon Rapoport. Mahwah, NJ, US: Lawrence Erlbaum Associates Publishers; 1999. pp. 387–410.

[44] Gong M, Baron J, Kunreuther H. Group cooperation under uncertainty. Journal of Risk and Uncertainty. 2009;39(3): 251–270. doi: 10.1007/s11166-009-9080-2

[45] Gong M, Baron J, Kunreuther H. Why do groups cooperate more than individuals to reduce risks? Theory and Decision. 2013;75(1): 101–116. doi: 10.1007/s11238-012-9318-3

[46] Jordan JJ, D.G. R, Arbesman S, Fowler JH, Christakis NA. Contagion of cooperation in static and fluid social networks. PLoS ONE. 2013;8(6): 1–10. doi: 10.1371/journal.pone.0066199 23840422 PMC3686805

[47] Au WT, Ngai MY. Effects of Group Size Uncertainty and Protocol of Play in a Common Pool Resource Dilemma. Group Processes & Intergroup Relations. 2003;6(3): 265–283. doi: 10.1177/13684302030063004

[48] Boosey L, Brookins P, Ryvkin D. Contests with group size uncertainty: Experimental evidence. Games and Economic Behavior. 2017;105: 212–29. doi: 10.1016/j.geb.2017.07.008

[49] de Kwaadsteniet EW, van Dijk E, Wit A, de Cremer D. “How many of us are there?”: Group size uncertainty and social value orientations in common resource dilemmas. Group Processes and Intergroup Relations. 2008;11(3): 387–399. doi: 10.1177/1368430208090649

[50] Hillenbrand A, Winter F. Volunteering under population uncertainty. Games and Economic Behavior. 2018;109:65–81. doi: 10.1016/j.geb.2017.12.009

[51] Mill W, Theelen MMP. Social value orientation and group size uncertainty in public good dilemmas. Journal of Behavioral and Experimental Economics. 2019;81: 19–38. doi: 10.1016/j.socec.2019.05.001

[52] Kim DG. Population uncertainty in voluntary contributions of public goods. Journal of Economic Behavior & Organization. 2018;145: 218–31. doi: 10.1016/j.jebo.2017.10.009

[53] Kahneman D. Thinking, fast and slow. New York, NY: Farrar, Straus and Giroux; 2011.

[54] Capraro V. The dual-process approach to human sociality: A review. PsyArXiv [preprint]. 2019. doi: 10.31234/osf.io/432yw

[55] de Neys W. On Dual- and Single-Process Models of Thinking. Perspectives on Psychological Science. 2021: 1–16. doi: 10.1177/1745691620964172 33621468

[56] de Neys W, Pennycook G. Logic, Fast and Slow: Advances in Dual-Process Theorizing. Current Directions in Psychological Science. 2019;28(5): 503–509. doi: 10.1177/0963721419855658

[57] Evans JSBT, Stanovich KE. Dual-Process Theories of Higher Cognition: Advancing the Debate. Perspectives on Psychological Science. 2013;8(3): 223–41. doi: 10.1177/1745691612460685 26172965

[58] Pennycook G, Neys W de, Evans JSBT, Stanovich KE, Thompson VA. The Mythical Dual-Process Typology. Trends in Cognitive Sciences. Elsevier Ltd. 2018;22(8): 667–668. doi: 10.1016/j.tics.2018.04.008 29937320

[59] Evans JSBT. Intuition and reasoning: A dual-process perspective. Psychological Inquiry. 2010;21(4): 313–326. doi: 10.1080/1047840x.2010.521057

[60] Kahneman D. A Perspective on Judgment and Choice: Mapping Bounded Rationality. American Psychologist. 2003; 58(9): 697–720. doi: 10.1037/0003-066X.58.9.697 14584987

[61] Rand DG, Greene JD, Nowak MA. Spontaneous giving and calculated greed. Nature. 2012;489(7416): 427–430. doi: 10.1038/nature11467 22996558

[62] Rand DG. Cooperation, Fast and Slow: Meta-Analytic Evidence for a Theory of Social Heuristics and Self-Interested Deliberation. Psychological Science. 2016; 27(9): 1192–1206. doi: 10.1177/0956797616654455 27422875

[63] Rand DG, Kraft-Todd GT. Reflection does not undermine self-interested prosociality. Frontiers in Behavioral Neuroscience. 2014;8. doi: 10.3389/fnbeh.2014.00300 25232309 PMC4153292

[64] Rand DG, Peysakhovich A, Kraft-Todd GT, Newman GE, Wurzbacher O, Nowak MA, et al. Social heuristics shape intuitive cooperation. Nature Communications. 2014;5(1): 1–12. doi: 10.1038/ncomms4677 24751464

[65] Bear A, Rand DG. Intuition, deliberation, and the evolution of cooperation. Proceedings of the National Academy of Sciences of the United States of America. 2016;113(4): 936–941. doi: 10.1073/pnas.1517780113 26755603 PMC4743833

[66] Jordan J, Peysakhovich A, Rand DG. Why we cooperate. In: Decety J, Wheatley T, editors. The moral brain: A multidisciplinary perspective. Cambridge, MA, US: Boston Review; 2015. pp. 87–101.

[67] Peysakhovich A, Rand DG. Habits of virtue: Creating norms of cooperation and defection in the laboratory. Management Science. 2016;62(3): 631–647. doi: 10.1287/mnsc.2015.2168

[68] Rand DG. Intuition, Deliberation, and Cooperation Further Meta-Analytic Evidence from 91 Experiments on Pure Cooperation. 2019. Retrieved from: https://paper s.ssrn.com/sol3/paper s.cfm?abstract_id = 33900 18.

[69] Capraro V, Cococcioni G. Social setting, intuition, and experience in laboratory experiments interact to shape cooperative decision-making. Proceedings of the Royal Society B: Biological Sciences. 2015;282(1811). doi: 10.1098/rspb.2015.0237 26156762 PMC4528537

[70] Isler O, Maule J, Starmer C. Is intuition really cooperative? Improved tests support the social heuristics hypothesis. PLoS ONE. 2018;13(1). doi: 10.1371/journal.pone.0190560 29304055 PMC5755815

[71] Everett JAC, Ingbretsen Z, Cushman F, Cikara M. Deliberation erodes cooperative behavior—Even towards competitive out-groups, even when using a control condition, and even when eliminating selection bias. Journal of Experimental Social Psychology. 2017;73: 76–81. doi: 10.1016/j.jesp.2017.06.014

[72] Rand DG, Newman GE, Wurzbacher OM. Social Context and the Dynamics of Cooperative Choice. Journal of Behavioral Decision Making. 2015;28(2): 159–66. doi: 10.1002/bdm.1837

[73] Cone J, Rand DG. Time pressure increases cooperation in competitively framed social dilemmas. PLoS ONE. 2014;9(12). doi: 10.1371/journal.pone.0115756 25551386 PMC4281206

[74] Capraro V, Cococcioni G. Rethinking spontaneous giving: Extreme time pressure and ego-depletion favor self-regarding reactions. Scientific Reports. 2016;6(1): 1–10. doi: 10.1038/s41598-016-0001-8 27251762 PMC4890119

[75] Goeschl T, Lohse J. Cooperation in public good games. Calculated or confused? European Economic Review. 2018;107: 185–203. doi: 10.1016/j.euroecorev.2018.05.007

[76] Lohse J. Smart or selfish–When smart guys finish nice. Journal of Behavioral and Experimental Economics. 2016;64:28–40. doi: 10.1016/j.socec.2016.04.002

[77] Bouwmeester S, Verkoeijen PPJL, Aczel B, Barbosa F, Bègue L, Brañas-Garza P, et al. Registered Replication Report: Rand, Greene, and Nowak (2012). Perspectives on Psychological Science. 2017;12(3): 527–42. doi: 10.1177/1745691617693624 28475467 PMC5453400

[78] Tinghög G, Andersson D, Bonn C, Böttiger H, Josephson C, Lundgren G, et al. Intuition and cooperation reconsidered. Nature. 2013; 498(7452): 427–430. doi: 10.1038/nature12194 23739429

[79] Verkoeijen PPJL, Bouwmeester S. Does intuition cause cooperation? PLoS ONE. 2014 May 6;9(5). doi: 10.1371/journal.pone.0096654 24801381 PMC4011763

[80] Bird BM, Geniole SN, Procyshyn TL, Ortiz TL, Carré JM, Watson N v. Effect of exogenous testosterone on cooperation depends on personality and time pressure. Neuropsychopharmacology. 2019;44(3): 538–545. doi: 10.1038/s41386-018-0220-8 30341408 PMC6333794

[81] Nishi A, Christakis NA, Evans AM, O’Malley AJ, Rand DG. Social Environment Shapes the Speed of Cooperation. Scientific Reports. 2016;6. doi: 10.1038/srep29622 27435940 PMC4951649

[82] Dal Bó P, Fréchette GR. The Evolution of Cooperation in Infinitely Repeated Games: Experimental Evidence. American Economic Review.2011;101(1): 411–429. doi: 10.1257/aer.101.1.411

[83] Fudenberg D, Rand DG, Dreber A. Slow to anger and fast to forgive: Cooperation in an uncertain world. American Economic Review. 2012;102(2): 720–749. doi: 10.1257/aer.102.2.720

[84] Kvarven A, Strømland E, Wollbrant C, Andersson D, Johannesson M, Tinghög G, et al. The intuitive cooperation hypothesis revisited: a meta-analytic examination of effect size and between-study heterogeneity. Journal of the Economic Science Association. 2020;6(1): 26–42. doi: 10.1007/s40881-020-00084-3

[85] Nishi A, Christakis NA, Rand DG. Cooperation, decision time, and culture: Online experiments with American and Indian participants. PLoS ONE. 2017;12(2). doi: 10.1371/journal.pone.0171252 28231296 PMC5322955

[86] Faul F, Erdfelder E, Buchner A, Lang A-G. Statistical power analyses using G*Power 3.1: Tests for correlation and regression analyses. Behavior Research Methods. 2009;41(4): 1149–1160. doi: 10.3758/BRM.41.4.1149 19897823

[87] Pleskac TJ, Wershbale A. Making Assessments While Taking Repeated Risks: A Pattern of Multiple Response Pathways. Journal of Experimental Psychology: General. 2014; 143(1): 142–162. doi: 10.1037/a0031106 23276285

[88] John OP, Srivastava S. The Big Five Trait Taxonomy: History, Measurement, and Theoretical Perspectives. In: Pervin L A., John O P, editors. Handbook of Personality: Theory and Research.1999;2: 102–138. New York, NY: Guilford Press.

[89] Brito Costa S, Bem-Haja P, Moisao A, Alberty A, Vicente Castro F, de Almeida H. Psychometric Properties of Portuguese Version of Big Five Inventory (BFI). International Journal of Developmental and Educational Psychology. 2016;1(2): 83–94. doi: 10.17060/ijodaep.2015.n2.v1.325

[90] Davis MH. Measuring individual differences in empathy: Evidence for a multidimensional approach. Journal of personality and social psychology. 1983; 44(1): 113–126. doi: 10.1037/0022-3514.44.1.113

[91] Limpo T, Alves RA, Catro SL. Medir a empatia: Adaptação portuguesa do Índice de Reactividade Interpessoal. Laboratório de Psicologia.2010; 8(2): 171–184. doi: 10.14417/lp.640

[92] Watson D, Clark LA, Tellegen A. Development and Validation of Brief Measures of Positive and Negative Affect: The PANAS Scales. Journal of Personality and Social Psychology. 1988; 54(6), 1063–1070. doi: 10.1037//0022-3514.54.6.1063 3397865

[93] Galinha IC, Pereira CR, Esteves F. Versão reduzida da escala portuguesa de afeto positivo e negativo-PANAS—VRP: Análise fatorial confirmatória e invariância temporal. Revista Psicologia. 2014; 28(1):53–65. http://hdl.handle.net/10451/11477.

[94] Schneider W, Eschman A, Zuccolotto A. E-prime User’s Guide. Pittsburgh: Psychology Software Tools; 2002.

[95] Fischbacher U. Z-Tree: Zurich toolbox for ready-made economic experiments. Experimental Economics. 2007;10(2):171–178. doi: 10.1007/s10683-006-9159-4

[96] R Core Team. R: A language and environment for statistical computing. R Foundation for Statistical Computing.Vienna: Austria; 2019 https://www.R-project.org/.

[97] Bates D. Fitting linear mixed models in R. R News. 2005; 5(1): 27–30. Available from: http://CRAN.R-project.org/doc/Rnews/.

[98] Evans AM, Dillon KD, Rand DG. Fast but not intuitive, slow but not reflective: Decision conflict drives reaction times in social dilemmas. Journal of Experimental Psychology: General. 2015;144(5): 951–966. doi: 10.1037/xge0000107 26413891

[99] Dal Bó P, Fréchette GR. On the Determinants of Cooperation in Infinitely Repeated Games: A Survey. Journal of Economic Literature. 2018;56(1):60–114. doi: 10.1257/jel.20160980

[100] Embrey M, Fréchette GR, Yuksel S. Cooperation in the Finitely Repeated Prisoner’s Dilemma. The Quarterly Journal of Economics. 2018;133(1):509–551. doi: 10.1093/qje/qjx033

[101] Evans AM, Rand DG. Cooperation and decision time. Current Opinion in Psychology.2019; 26:67–71. doi: 10.1016/j.copsyc.2018.05.007 29879640

[102] Gallotti R., Grujić J. A quantitative description of the transition between intuitive altruism and rational deliberation in iterated Prisoner’s Dilemma experiments. Scientific reports. 2019; 9(1):1–11. doi: 10.1038/s41598-018-37186-2 31745100 PMC6864093

[103] Chen X-P, Wing A, Au T, Komorita SS. Sequential Choice in a Step-Level Public Goods Dilemma: The Effects of Criticality and Uncertainty. Organizational Behavior and Human Decision Processes. 1996; 65(1): 37–47. doi: 10.1006/obhd.1996.0003

[104] Kappes A, Nussberger A-M, Siegel JZ, Rutledge RB, Crockett MJ. Social uncertainty is heterogeneous and sometimes valuable. Nature Human Behaviour. 2019;3(8):764. doi: 10.1038/s41562-019-0662-y 31358972

[105] Kappes A, Nussberger AM, Faber NS, Kahane G, Savulescu J, Crockett MJ. Uncertainty about the impact of social decisions increases prosocial behaviour.Nature Human Behaviour. 2018; 2(8): 573–580. doi: 10.1038/s41562-018-0372-x 31209312 PMC6639117

[106] Stagnaro MN, Arechar AA, Rand DG. From good institutions to generous citizens: Top-down incentives to cooperate promote subsequent prosociality but not norm enforcement. Cognition. 2017;167:212–254. doi: 10.1016/j.cognition.2017.01.017 28249658 PMC5875418

[107] Kahneman D, Tversky A. Discussion On the interpretation of intuitive probability: A reply to Jonathan Cohen. Cognition. 1979; 7(4), 409–411. doi: 10.1016/0010-0277(79)90024-6

[108] Mantilla C. Environmental uncertainty in commons dilemmas: A survey of experimental research. International Journal of the Commons. 2018;12(2):300–329. doi: 10.18352/ijc.857

