# Supplementary figures and images for: A dual-process approach to cooperative decision-making under uncertainty
Source: PLoS One. 2022 Mar 22;17(3):e0265759. doi: 10.1371/journal.pone.0265759 (PMC8939828; doi:10.1371/journal.pone.0265759)

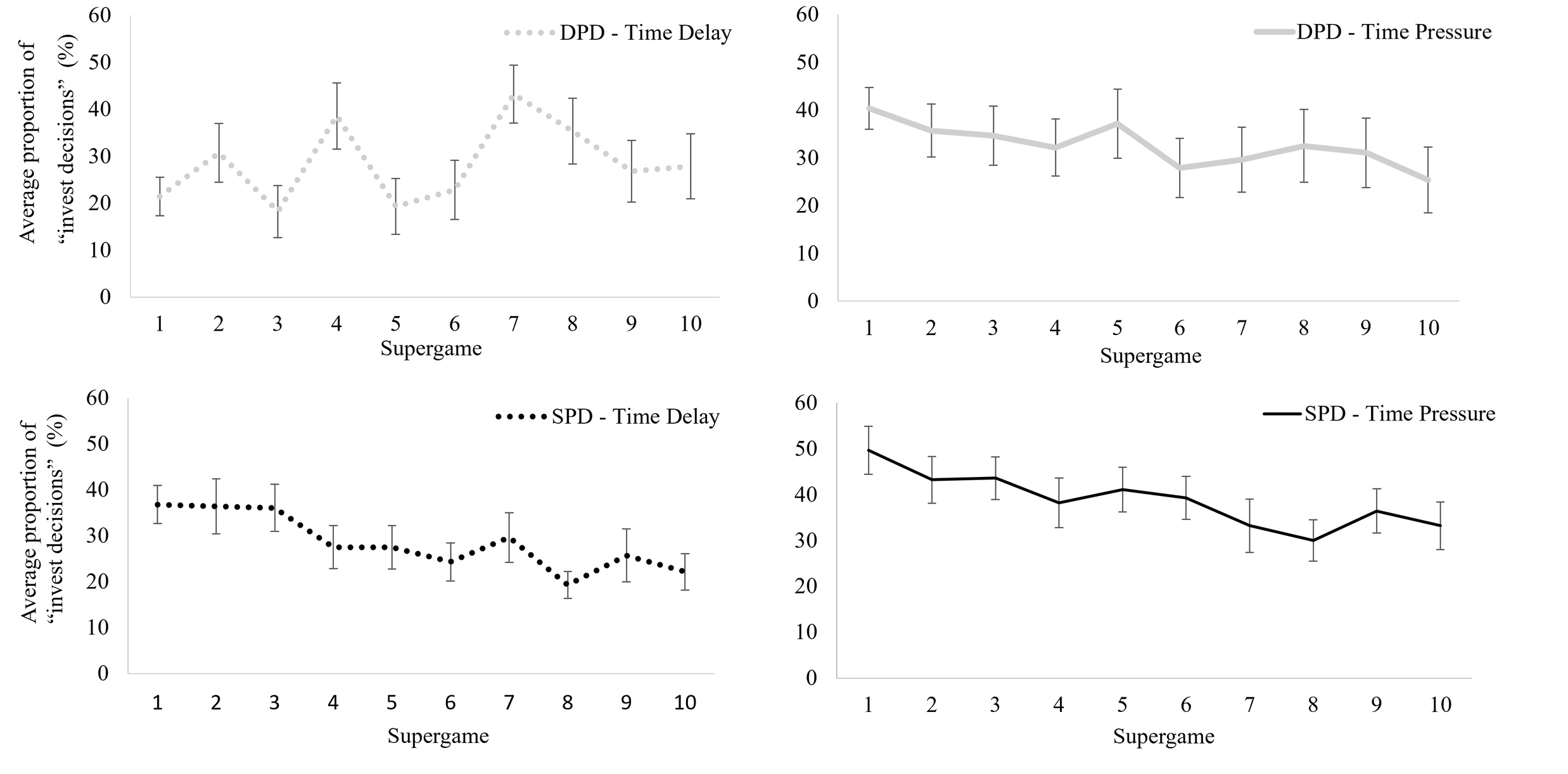

Supplement: S1 Fig — n = 28 participants in each graphic presented. Error bars indicate standard errors of the mean. (TIF) [file pone.0265759.s001.tif]

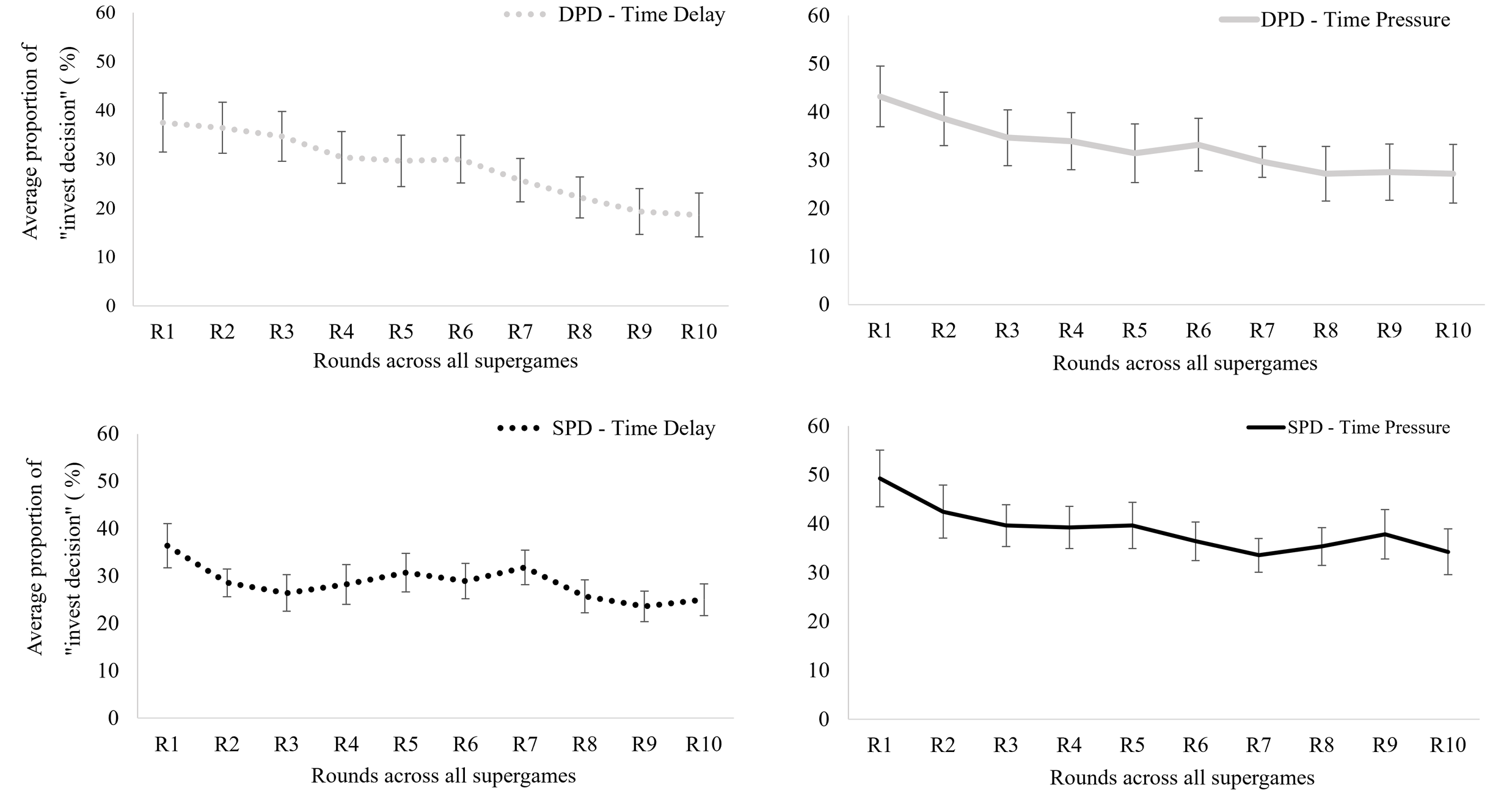

Supplement: S2 Fig — n = 28 participants in each graphic presented. Error bars indicate standard errors of the mean. (TIF) [file pone.0265759.s002.tif]
