# Supplementary material for: A dual-process approach to cooperative decision-making under uncertainty
Source: PLoS One. 2022 Mar 22;17(3):e0265759. doi: 10.1371/journal.pone.0265759 (PMC8939828; doi:10.1371/journal.pone.0265759)
Supplement: S1 Table — (DOCX) [file pone.0265759.s003.docx]

# Instructions

(Translated from Portuguese)

## General Instructions

Thank you for participating in this experiment!

You have received 0.5 credit courses for participating. In this study, we recruit students to resolve several problem-solving challenges!

Initially, you will play a game in which the outcomes of your decisions depend not only on what you choose to do but also on what your counterpart does.

Your counterpart in this game will be the other participants present in the experimental room. Still, you will not learn who the other participants are, and they will not know about your identity.

You will play multiple sessions. For example, participants will play against the same counterpart in one Supergame (10 rounds), then switch counterparts in the next Supergame. At the beginning of each supergame, all participants will be given 1500 points.

In this game, we simulate a situation in which it is essential to avoid more significant losses. Therefore, each of you will decide independently whether or not to invest some of your initial 1500 points to lose as few points as possible.

Please keep in mind that the experiment cannot continue until all participants make their

decisions. You will not know the decision your counterpart has made until the end of the round. Before starting the next round, you will be given feedback on what you and

your counterpart did in this round and the status of your points.

The score at the end of each round is essential since it will later determine the reward you might receive. Specifically, two participants (of all who are participating in the experiment) will be randomly selected to receive a cash voucher of a value proportional to the points from one random supergame (the exchange rate is 1 point for 0.03 cents - 1500 points = 45 euro). The amount of points you keep at the end of each supergame is your payoff for that supergame. The final payoffs might depend on one random Supergame. To illustrate, suppose that someone is randomly chosen to be paid for their Supergame 4. Suppose at the end of Supergame 4 they have 900 points. At the end of the experiment, this person will receive 27 euros.

Raise your hand if you have any questions regarding the experiment. We will then come directly to your place.

Otherwise, please press the "*continue*" button to read the following instructions regarding the game's rules.

## Rules of the game (SPD or DPD)

In each round, you have to choose one out of two alternative decisions, INVEST and NOT INVEST. Depending on your opponent's decision, your payoff is then determined according to the table below. You find your own payoff at the first position of each item and the payoff of your opponent in the second position.

-----------------------------------[Stochastic Prisoner's Dilemma (SPD)]----------------------------------

- If both you and the other player choose to INVEST, then it will cost each of you 45 points.
- If one of you chooses to INVEST and the other chooses NOT INVEST.

In that case,

- the one who chooses INVEST loses 45 points and has a 20% probability of losing additional 100 points. That is, the investor has a 20% probability of losing 145 points and an 80% probability of losing 45 points,

and,

- the other who chooses NOT INVEST has a 40% probability of losing 100 points and a 60% probability of not losing any points.
- If both of you choose NOT INVEST, then each of you will have a 52% probability of losing 100 points and a 48% of not losing any points.

The table illustrates the four possible outcomes:

|  | Your counterpart | | | |
| --- | --- | --- | --- | --- |
| You |  | Invest | Not Invest |  |
|  | Invest | -45; - 45 | - 20% lose 145, 80% lose 45; - 40% lose 100, 60% lose 0 |  |
|  | Not Invest | - 40% lose 100, 60% lose 0; - 20% lose 145, 80% lose 45 | - 52% lose 100, 48% lose 0; - 52% lose 100, 48% lose 0 |  |

-------------------------------------------------------------------------------------------------------------------------------

-----------------------------------[Deterministic Prisoner's Dilemma (DPD)]-----------------------------

- If both you and the other player choose to INVEST, then it will cost each of you 45 points.
- If one of you chooses to INVEST and the other chooses NOT INVEST.

In that case,

– the one who chooses INVEST loses 65 points,

and,

– the other who chooses NOT INVEST loses 40 points.

- If both of you choose NOT INVEST, then it will cost each of you 52 points.

The table illustrates the four possible outcomes:

|  | Player 2 | | |
| --- | --- | --- | --- |
| Player 1 |  | Invest | Not Invest |
|  | Invest | -45; -45 | -65; -40 |
|  | Not Invest | -40; -65 | -52; -52 |

---------------------------------------------------------------------------------------------------------------------------The information present in the table will appear in every round. Please raise your hand if you have any questions. Otherwise, please click on "*Continue*" to read the final instructions.

## Instructions on time

[Time pressure condition] Please make your decision as quickly as possible. You must make your decision in less than 10 seconds!

[Time delay condition] Please carefully consider you decision. You must wait and think for at least 10 seconds before making your decision.

Please click "*Continue*" when you are ready to start the experiment.
