# Supplementary material for: A dual-process approach to cooperative decision-making under uncertainty
Source: PLoS One. 2022 Mar 22;17(3):e0265759. doi: 10.1371/journal.pone.0265759 (PMC8939828; doi:10.1371/journal.pone.0265759)
Supplement: S1 Appendix — The effects of individual differences on cooperation. (DOCX) [file pone.0265759.s004.docx]

**Supplementary Notes**

**Additional results regarding the individual differences on the average proportion of cooperation**

We conducted a forward and backward stepwise multiple linear regression to evaluate if individual differences influence the average cooperative behavior. The individual differences that we analyzed were: i) personality factors from the Big Five Inventory; ii) the four dimensions from the empathy scale; iii) the positive and negative affect previous and after the experiment; iv) the individual risk-taking propensity calculated by the average number of pumps for all unexploded balloons; v) sociodemographic variables: age, sex, socioeconomic status, and given that all participants are from a Psychology major, the year they were enrolled; vi) and game-related questions: how much they rated their day-to-day basis cooperation with the other players, how much participants rate they are close (proximity) with the other players, the difficulty experienced during the execution of the task, understanding the task and the instructions, how stressful was the execution of the task, and rate how pleasant was the performance of the task.

Conducting the most parsimonious model (the model with the lowest AIC - Akaike Information Criterion - AIC= -369.71), we found that some of the individual difference measures were statistically significant predictors of the average proportion of cooperation *F*(12,99) = 5.35, *p* < .001, *R²* =.394. Table 1 reveals that in the second model, the neuroticism trait of personality is a significant negative predictor of the average proportion of cooperation (*β* = -.013, *t* = -3.686, *p* < .001, OR = .99, 95 % CI = .98, 99). At the beginning of the experiment, the negative affect revealed a significant effect (*β* = .031, *t* = 3.13, *p* = .002, OR = 1.03, 95% CI=1.01,1.05). At the end of the experiment, the positive affect revealed a significant effect (*β* = .020, *t* = .006, *p* = < .001, OR = 1.02, 95% CI = 1.01, 1.03) on the average proportion of cooperation. Additionally, we applied two independent t-tests to compare positive affect previous and after the experiment and found a statistical difference *t*(111) = -5,259, *p* ≤ .001. Positive affect improved at the end of the experimental task. We found no statistical differences between the negative affect previous and after the experiment ends *t*(111) = .729, *p* = .468. The model revealed a marginally significant effect on the average number of pumps in the BART task (β = .003, *t* = .001, *p* = .061, OR = 1.00, 95 % = 1.00, 1.01).

Regarding the sociodemografique variables, the socio-economic value reveals to be a statistically significant predictor of the average probability of cooperation. Participants who report having a medium-high socio-economic value (n = 15) have a higher average probability of cooperation ( β = .208, *t* = .058, *p* < .001, OR = 1.23, 95 %= 1.10, 1.38). Two questions regarding the execution of the task reveal to be a statistically significant positive predictor of the average probability of cooperation: how pleasant were the execution of the task (β = .029, *t* = 2.559, *p* = .012, OR = 1.03, 95% = 1.01, 1.05) and the difficulty experienced (β = .029, *t* = 2.220, p = .029, OR =1.03, 95% = 1.00, 1.06). Moreover, the question regarding how stressful the task's execution is, revealed to be a marginally significant predictor (β = .024, *t* = 1.935, *p* = .056, OR = 1.02, 95% CI= 1.00,1.05).

**Table 1. Multiple linear regression on the average cooperation rate**

|  | *Model 1* | | | *Model 2* | | |
| --- | --- | --- | --- | --- | --- | --- |
|  | *β (SE)* | *t* | *p* | *β (SE)* | *t* | *p* |
| Intercept | -.50(.42) |  |  | -.09(.15) |  |  |
| Positive Affect | -.01(.01) | -1.81 | .074 | .03(.01) | 3.13 | .002** |
| Negative Affect | .03(.01) | 1.94 | .056 | -.01(.01) | -1.74 | .085 |
| End_Pos Affect | .02(.01) | 2.73 | .008 | .02(.01) | 3.29 | .001*** |
| End_Neg Affect | .00(.02) | .22 | .828 |  |  |  |
| BFI_neuroticism | -.01(.01) | -1.97 | .053 | -.01(.00) | -3.69 | <.001*** |
| BFI_extraversion | .00(.01) | .82 | .413 |  |  |  |
| BFI_agreeableness | -.00(.00) | -.64 | .525 |  |  |  |
| BFI_conscientiousness | .00(.00) | .61 | .546 |  |  |  |
| BFI_openness | -.00(.00) | -.18 | .855 |  |  |  |
| Empathy_PT | -.02(.01) | 1.70 | .094 |  |  |  |
| Empathy_EC | -.01(.01) | -.91 | .368 |  |  |  |
| Empathy_PD | .01(.01) | .81 | .420 |  |  |  |
| Empathy_F | -.00(.01) | -.31 | .760 |  |  |  |
| BART-Average_Pump | .00(.00) | 1.88 | .064 | .00(.00) | 1.89 | .061 |
| Age | -.00(.01) | -.04 | .970 |  |  |  |
| Gender | .10(.06) | 1.76 | .083 |  |  |  |
| SES Level 3 | .04(.04) | .81 | .419 | .04(.04) | 1.03 | .305 |
| SES Level 4 | .22(.06) | 3.38 | .001 | .21(.06) | 3.56 | <.001*** |
| Year of school | .01(.02) | .35 | .725 |  |  |  |
| Cooperate | -.01(.01) | -.78 | .437 | -.01(.01) | -1.35 | .179 |
| Proximity | -.00(.02) | -.04 | .971 |  |  |  |
| Difficulty experienced | .03(.02) | 2.21 | .030 | .03(.01) | 2.22 | .029* |
| Understanding of the task | -.02(.02) | -.74 | .460 |  |  |  |
| Stressful | .02(.01) | 1.54 | .127 | .02(.01) | 1.94 | .056 |
| Pleasant | .03(.01) | 2.14 | .036 | .03(.01) | 2.56 | .012* |
| N | 112 |  |  | 112 |  |  |
| *F(df)* | 2.53(25,86) |  |  | 5.35(12,99) |  |  |
| Adj *R*² | .26 |  |  | .32 |  |  |

**Discussion**

Following the suggestion of previous research [1] in our experiment, we also explored the degree to which individual determinants affect cooperative behavior. Specifically, we explore if the individual risk-taking propensity predicts the previous task's cooperation rate. The score in the BART task was computed by averaging the number of pumps for all unexploded balloons [2, 3]. A higher BART score indicated a higher level of risk‐taking [2]. Results demonstrated that the more risk-taking propensity an individual presents, the more he cooperates in the previous task. We find a marginally significant effect that suggests that individuals with a risk-taking propensity would cooperate more. Literature supports these findings, revealing that risk-seeking individuals were more cooperative than risk-averse individuals [4, 5, 6].

In our study regarding personality traits, lower levels of neuroticism predict the likelihood of cooperation in the prisoner's dilemma. [Neuroticism](https://www.sciencedirect.com/topics/psychology/neuroticism) is defined as the trait disposition to experience negative effects. It involves vulnerability to stress and the experience of negative emotions (e.g., anxiety, hostility, and depression) [7]. The result found is consistent with previous research revealing that lower neuroticism levels predicted higher cooperation levels in the incentivized prisoner's dilemma game [8]. Previous findings also report a positive but not significant effect between neuroticism and cooperation in a hypothetical game. The negative effect of neuroticism on cooperation was also found in other social dilemma games [9,10,11]. A study revealed that neuroticism is the best predictor of risk-taking among personality factors in a loss domain [12]. Additionally, we do not find an effect of the other personality traits on predicting cooperative behaviors: openness to experience [8], extroversion [13], and agreeableness [14] (for a review, see [6, 11]).

Relatively to mood effects, our results demonstrate an increase in the positive affect reported at the end of the experiment, compared with the beginning. Also, the positive affect at the end of the game task is a significant predictor of the average cooperation rate. The more positive affect reported at the end of the experiment, the higher the average cooperation rate. Previous research suggests that a positive mood increases cooperative and prosocial behavior [15,16,17].

Most studies have explored the relationship between mood and prosocial behavior by inducing people to be in a positive, negative, and neutral mood [15,16,17]. Our study only asks people to rate their current mood to control their dispositional mood at the time of the experience. Interestingly we found that the positive affect reported at the beginning of the game task was a significant negative predictor of the average cooperation rate. It appears that individuals who report more positive affect at the beginning have a decrease in the average cooperation rate. Additionally, we found that the negative affect reported at the beginning of the experiment is a significant preditor, which means that individuals who report more negative affect at the beginning of the experiment have a higher average cooperation rate.

Regarding sociodemographic variables, the socioeconomic status reveals to be a significant predictor of the individual cooperation mean, revealing that participants who report a medium-high socioeconomic status cooperate more. Although few students reported this status (*n* =18), most indicated a medium socioeconomic status (*n* = 64).

Additionally, two game-related questions were revealed to be significant predictors of the individual average propensity to cooperate. The difficulty experienced and the pleasure experienced during the task were revealed to be significant positive predictors. The more difficult participants evaluated the task and the more they rated the task as pleasant, the more they cooperated.

**Supplementary References**

1.  Alós-Ferrer C, Garagnani M. The cognitive foundations of cooperation. Journal of Economic Behavior and Organization. 2020;175: 71–85. doi: 10.1016/j.jebo.2020.04.019

2. Liu Z, Liu T, Mu S.Gender differences in the effects of competition and cooperation on risk‐taking under ambiguity. PsyCh journal. 2021;10:374–383.doi:10.1002/pchj.419

3. Lejuez CW, Read JP, Kahler CW, Richards JB, Ramsey SE, Stuart GL, Strong DR, Brown RA. Evaluation of a behavioral measure of risk-taking: The balloon analogue risk task (BART). Journal of Experimental Psychology: Applied. 2002;8(2):75–84.doi:10.1037/1076-898X.8.2.75

4. Fung JM, Au WT, Hu W, Shi K. Effect of risk orientation on cooperation and decision process in public goods dilemma.Group Processes & Intergroup Relations.2012;15(6):791-803.doi:10.1177/1368430212448443

5. Parks C D.Risk Preference As a Predictor of Cooperation in a Social Dilemma.In: Suleiman R , Budescu D V, Fischer I, Messick D M, editors. Contemporary psychological research on social dilemmas.New York, NY, US: Cambridge University Press; 2004. pp.315–331

6. Thielmann I, Spadaro G, Balliet D. Personality and prosocial behavior: A theoretical framework and meta-analysis. Psychological Bulletin.2020;146(1):30-90.doi:10.1037/bul0000217

7. McCrae R R, Costa P T.Validation of the five-factor model of personality across instruments and observers.Journal of Personality and Social Psychology.1987; 52(1): 81–90.doi:10.1037/0022-3514.52.1.81

8. Lönnqvist J E, Verkasalo M, Walkowitz G. It pays to pay–Big Five personality influences on co-operative behavior in an incentivized and hypothetical prisoner's dilemma game. Personality and Individual Differences.2011;50(2):300-304.doi:10.1016/j.paid.2010.10.009

9. Fahr R, Irlenbusch B.Identifying personality traits to enhance trust between organisations: An experimental approach. Managerial and Decision Economics.2008;29(6):469-487.doi:10.1002/mde.1415

10. Müller J, Schwieren C. Can personality explain what is underlying women's unwillingness to compete?.Journal of Economic Psychology.2012;33(3): 448-460. doi:10.1016/j.joep.2011.12.005

11. Zhao K, Smillie L D.The role of interpersonal traits in social decision making:Exploring sources of behavioral heterogeneity in economic games. Personality and Social Psychology Review.2015;19(3): 277-302.doi:10.1177/1088868314553709

12. Lauriola M, Levin I P.Personality traits and risky decision-making in a controlled experimental task: An exploratory study. Personality and individual differences.2001;31(2):215-226. doi:10.1016/S0191-8869(00)00130-6

13. Hirsh J B, Peterson J B.Extraversion, neuroticism, and the prisoner's dilemma. Personality and Individual Differences.2009;46(2): 254-256.doi:10.1016/j.paid.2008.10.006

14. Kagel J, McGee P.Personality and cooperation in finitely repeated prisoner's dilemma games. Economics Letters.2014;124(2):274-277.doi:10.1016/j.econlet.2014.05.034

15. Hertel G, Neuhof J, Theuer T, Kerr N L. Mood effects on cooperation in small groups: Does positive mood simply lead to more cooperation?. Cognition & emotion.2000; 14(4): 441-472.doi:10.1080/026999300402754

16. Kirchsteiger G, Rigotti L, Rustichini A. Your morals might be your moods. Journal of Economic Behavior & Organization.2006;59(2):155-172. doi:10.1016/j.jebo.2004.07.004

17. Lount R B.The impact of positive mood on trust in interpersonal and intergroup interactions. Journal of personality and social psychology. 2010;98(3): 420-433. doi:10.1037/a0017344
